# *Miyalachnus*—A New Lachninae Aphid Genus from Japan (Insecta, Hemiptera, Aphididae)

**DOI:** 10.3390/insects15030203

**Published:** 2024-03-18

**Authors:** Mariusz Kanturski, Yerim Lee

**Affiliations:** 1 Institute of Biology, Biotechnology and Environmental Protection, Faculty of Natural Sciences, University of Silesia in Katowice, Bankowa 9, 40-007 Katowice, Poland; 2 Department of Biological Sciences, Kunsan National University, 558 Daehak-ro, Naun 2(i)-dong, Gunsan-si 54150, Jeollabuk-do, Republic of Korea; ecoylls@gmail.com

**Keywords:** Lachninae, morphology, *Prunus*, *Pyrolachnus*, SEM, sensilla, Tuberolachnini

## Abstract

**Simple Summary:**

This publication describes a new Lachninae aphid genus and species (Insecta, Hemiptera: Aphididae)—*Miyalachnus*—from Japan. This genus is characterized by unique and outstanding morphological features on the legs and antennae and can be easily recognized within the remaining Lachninae genera. We provided a detailed scanning electron microscopy study on the general morphology and sensilla of the viviparous and sexual generations. We presented the biology of the genus with descriptions of all available morphs.

**Abstract:**

The tribe Tuberolachnini within the Lachninae (Hemiptera: Aphididae) is particularly intriguing due to its morphological traits and various ecological associations. Among the genera within this group, *Pyrolachnus* stands out as relatively understudied. Currently, only one species, *Pyrolachnus imbricatus nipponicus* Sorin, 2011, is known from Japan, distinguished by its distinctive characteristics. Through meticulous morphological analyses, we introduce a novel Lachninae genus, *Miyalachnus* **gen**. **nov**., associated with *Cerasus* and *Prunus* spp. (Rosaceae) in Japan. This new genus accommodates *P*. *imbricatus nipponicus*, now recognized as *Miyalachnus nipponicus* (Sorin, 2011) **comb**. **nov**. Additionally, we present a second species within this genus, *Miyalachnus sorini* **sp**. **nov**., along with comprehensive SEM morphological examination and insights into its biology. Our study describes in detail the morphological characteristics of both viviparous and bisexual generations of *Miyalachnus*, as well as their relationships with related genera.

## 1. Introduction

The subfamily Lachninae Herrich-Schaeffer, 1854 is among the 23 subfamilies of Aphididae [1], characterized by its large body size and capable of feeding on both green and woody parts of coniferous and deciduous plants [2]. Most aphids within the Lachninae inhabit trees and shrubs, with three genera from the tribe Tramini Herrich-Schaeffer, 1854, also found on the roots of herbaceous plants [3,4,5,6,7,8,9].

Tuberolachnini was established by Oestlund [10] but without the broad acceptance of aphidologists. Several authors who presented different Lachninae classification proposals never used Tuberolachnini as an independent group [11,12,13,14,15,16]. Only Mamontova [17,18,19,20] treated Tuberolachnini as one of the tribes within Lachninae. With the development of molecular methods in aphid taxonomy, Normark [21] first tested the relationships within Lachninae with *Nippolachnus piri* Matsumura and *Tuberolachnus salignus* (Gmelin, 1790), forming an independent clade. This result has been confirmed by Chen et al. [2], along with *Pyrolachnus* Basu and Hille Ris Lambers, 1968, which also nested within Tuberolachnini. Further, Chen et al. [2] transferred *Sinolachnus* Hille Ris Lambers, 1956 into Tuberolachnini but without any morphological or molecular evidence. Those authors, moreover, included *Neonippolachnus* Shinji, 1924 as a member of Tuberolachnini. In recent years, Kanturski et al. [22] started the work on the taxonomy, morphology, and phylogeny of Tuberolachnini. Kanturski et al. [23] revised the genus *Sinolachnus* and, as a result of detailed morphological analyses with remaining Lachninae, transferred this genus to the tribe Tramini. During the revision of the genus *Nippolachnus* Matsumura, 1917, Kanturski et al. [24] erected a new genus within Tuberolachnini—*Indolachnus* Kanturski, Lee, Koszela and Lee, 2024 and proposed *Neonippolachnus* as a synonym of *Nippolachnus*. The results of Kanturski et al. [22,23,24] clearly showed that Tuberolachnini is a group that needs taxonomical and phylogenetic analyses. *Pyrolachnus* currently comprises four known species, whose representatives are found on fruit trees belonging to the Rosaceae family: *Eriobotrya* (*Pyrolachnus macroconus* Zhang and Zhong, 1982 and *Pyrolachnus pyri* (Buckton, 1899)), *Malus*, *Pyrus* (*P. pyri*), and *Prunus* (*Pyrolachnus imbricatus* David, Narayanan and Rajasingh, 1971) [25]. One species—*P. macrorhinarious* Tao, 1989—was described from an alate viviparous female; so, its host plant is unknown. *Pyrolachnus imbricatus* was initially described from *Prunus cornuta* in India by David et al. [25]. Subsequently, Sorin [26] described a new subspecies, *P*. *imbricatus nipponicus*, based on specimens collected from *Prunus yedoensis* in Japan. In the Remarks section, Sorin highlighted several clear differences such as a significantly smaller body size, more secondary setae on the ultimate rostrum segment, and additional setae on the eighth abdominal segment. Moreover, in the description, Sorin noted some unusual characteristics for *Pyrolachnus* species, such as a spinulose dorsum and a unique arrangement of accessory rhinaria on the last antennal segment. Blackman and Eastop [27] mentioned that the described population from Japan, in addition to the much smaller body size, exhibits other significant differences that may “justify full species status”.

Due to the aforementioned morphological differences of *P*. *imbricatus nipponicus* from other *Pyrolachnus* species and the status of *Sinolachnus* as pointed out by Kanturski et al. [23], we conducted detailed analyses of the morphology and sensilla of *P*. *imbricatus nipponicus*. As a result of the remarkable morphological differences and characters, we propose a new genus, *Miyalachnus*, to accommodate *P*. *imbricatus nipponicus* Sorin, 2011 as *Miyalachnus nipponicus* (Sorin, 2011). Additionally, we describe a second species within this genus, presenting all available morphs, detailed SEM investigations, and discussion of their biology and relations to other Lachninae genera.

This study forms part of the revision of the genus *Pyrolachnus* and phylogenetic analyses of the tribe Tuberolachnini.

## 2. Materials and Methods

### 2.1. Study Material, Light Microscopy, and Abbreviations

The samples were collected in the field and directly preserved in 70% ethanol for further analysis. In the laboratory, the ethanol-preserved samples were added to 10% alcohol-fixed samples, which were then added to 10% KOH and heated for several minutes. After maceration of the tissues, the embryos were removed and the samples were transferred into chloral phenol for one hour, and after that time, they were transferred to chloral hydrate. From chloral hydrate, the aphids were put into a Faure–Berlese solution and dried in an incubator for about seven days at 40 °C. All existing and available slides of representatives of Tuberolachnini and Lachninae used for this study were examined using a Leica DM 3000 LED light microscope and photographed with a Leica MC 190 HD camera. The measurements were made according to Ilharco and van Harten [28] and Blackman and Eastop [29]. Measurements are given in millimeters. Actual host plant names are given according to World Flora Online [30]. Final figure processing was conducted using PhotoScape 3.7 (photoscape.org, accessed on 17 May 2020) and IrfanView 64 (irfanview.com, accessed on 17 May 2020). The following Tuberolachnini and Lachninae taxa were examined for this study: *Indolachnus himalayensis* (van der Goot, 1917), *Nippolachnus piri* Matsumura, 1917, *N. micromeli* Shinji, 1924, *Pyrolachnus mbricatus* David, Narayanan and Rajasingh, 1972, *P. macroconus* Zhang and Zhong, 1982, *P. pyri* (Buckton, 1899), *Tuberolachnus* (*Tuberolachnus*) *salignus* (Gmelin, 1790), *Tuberolachnus* (*Tuberolachniella*) *scleratus* Hille Ris Lambers and Basu, 1966, *T*. (*T*.) *macrotuberculatus* Yang, Qiao, and Zhang, 2005, *Sinolachnus niitakayamensis* (Takahashi, 1925), *S. yushanensis* Kanturski, Yeh, and Lee, 2022, *Eotrama moerickei* Hille Ris Lambers, 1969, *Protrama radicis* (Kaltenbach, 1843), *P. ranunculi* (Del Guercio, 1909), *P. flavescens* (Koch, 1857), *Trama rara* Mordvilko, 1909, *T. troglodytes* von Heyden, 1837 *Lachnus roboris* (Linnaeus, 1758), *L. tatakaensis* Takahashi, 1937, *L. tropicalis* (van der Goot, 1916), *Maculolachnus submacula* (Walker, 1848), *Longistigma caryae* (Harris, 1841), *Stomaphis* (*Stomaphis*) *quercus* (Linnaeus, 1758), and *S*. (*Parastomaphis*) *graffii* Cholodkovsky, 1894.

The following abbreviations are used in this paper:

BL—body length (from the anterior border of the head to the end of the cauda); Max W—greatest body width across the middle of the abdomen; HW—greatest head width across compound eyes; ANT—antennae or their lengths; ANT I, II, III, IV, V, and VI—antennal segments I, II, III, IV, V, and VI or their lengths (ratios between antennal segments are simply given as, e.g., ‘VI:III’); LS—length of the longest setae of ANT III; BD III—basal articular diameter of ANT III; BASE—basal part of the last antennal segment or its length; PT—processus terminalis of the last antennal segment or its length; URS—ultimate rostrum segments (IV + V) or their length; III FEMORA—hind femur; III TIBIA—hind tibia; FT I—first segment of fore tarsus; MT I—first segment of middle tarsus; HT I—first segment of hind tarsus; HT Ib—basal length of HT I; HT Id—dorsal length of HT I; HT Iv—ventral length of HT I; HT Ii—intersegmental length of HT I; HT II—second segment of the hind tarsus or its length; ABD I–VIII—abdominal tergite I–VIII, SIPH—siphunculi; Fx—fundatrix (stem mother); apt—apterous viviparous female; al—alate viviparous female; ♂—male; ♀—sexual (oviparous) female.

Depositories of Lachninae andTuberolachnini material studied:

DZUS—Zoology Research Team, Faculty of Natural Sciences, University of Silesia in 

Katowice, Katowice, Poland.

MNHN—Muséum national d’Histoire naturelle, Paris, France.

NHMUK—Natural History Museum in London, London, UK.

NIAES—National Institute for Agro-Environmental Science, Kannon-dai, Tsukuba

City, Ibaraki Prefecture, Japan.

TARI—Taiwan Agricultural Research Institute, Taichung City, Taiwan.

VCK—Vidyasagar College, Kolkata, India.

The holotype of the new species will be deposited at DZUS. Paratypes will be also deposited at NIAES, MNHN, and NHMUK.

### 2.2. Scanning Electron Microscopy

Field-collected individuals of viviparous females, sexuales (oviparous females and males), and eggs of the new genus were preserved in 2.5% glutaraldehyde and 80% ethanol for several days. In the laboratory, the preserved samples were dehydrated in an ethanol series (80%, 90%, and 96%) and absolute ethanol two times for 10 min each. Dehydrated samples were transferred to chloroform from absolute ethanol for 24–36 h. After dehydration and cleaning, the samples were dried using the Leica EM CPD 300 auto-critical point dryer (Leica Microsystems, Vienna, Austria). Dry aphid samples were mounted on aluminum stubs with double-sided adhesive carbon tape and coated with a 30-nm layer of gold in a Quorum 150 T ES Plus sputter coater (Quorum Technologies Ltd., Laughton, East Sussex, UK). The Hitachi SU8010 field emission scanning electron microscope FESEM (Hitachi High-Technologies Corporation, Tokyo, Japan) imaged the samples at 7 and 10 kV accelerating voltage with a secondary electron detector (ESD). Three-dimensional (3D) reconstructions of the body surface of the new genus were made using a Phenom XL scanning electron microscope (Phenom-World B.V., Eindhoven, The Netherlands) at 15 kV accelerating voltage, using a secondary electron detector with Phenom 3D Roughness.

Reconstruction Software: Figure processing for this manuscript was performed using PhotoScape 3.7 (photoscape.org, accessed on 17 May 2020) and the IrfanView 64 software (irfanview.com, accessed on 17 May 2020).

## 3. Results

### 3.1. Taxonomy

Genus *Miyalachnus* **gen**. **nov**.

Type species: *Miyalachnus sorini* **sp**. **nov**., here designated

 

***Diagnosis:***

**Figure 1:**
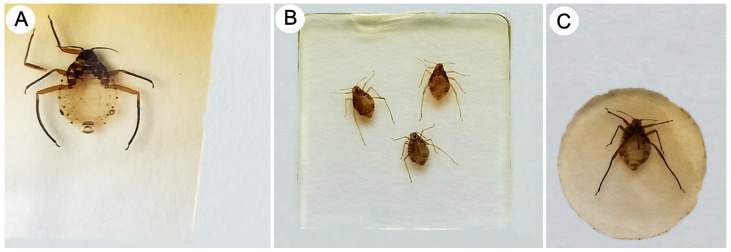
Body size comparison between the biggest *Pyrolachnus* species (*Pyrolachnus* sp.) (**A**), *Miyalachnus nipponicus* (**B**), and the smallest *Pyrolachnus* species (*P. pyri*) (**C**).

**Figure 2:**
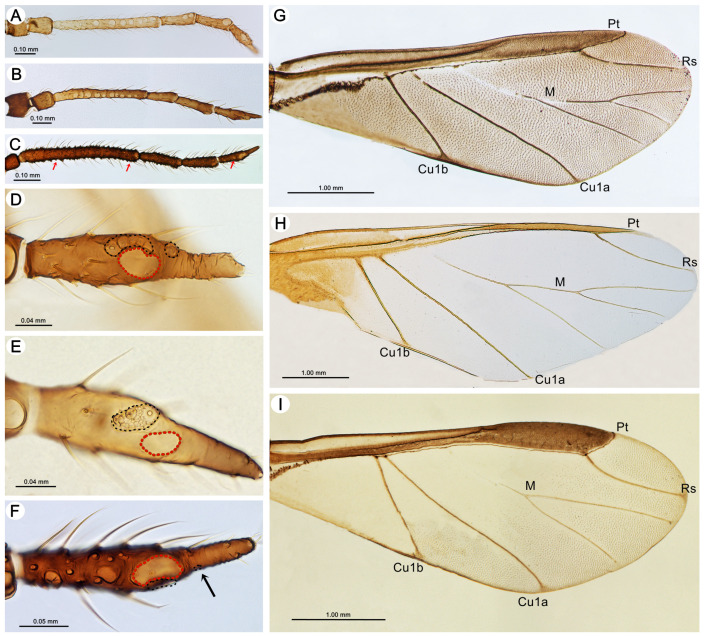
The most significant differences between alate viviparous females of *Miyalachnus* and the most similar genera, such as *Pyrolachnus* (Tuberolachnini) and *Sinolachnus* representatives on woody Rosaceae, are shown: (**A**) ANT of *M. nipponicus* with big, rounded secondary rhinaria on ANT III and IV, (**B**) ANT of *P. imbricatus* with big, rounded secondary rhinaria on ANT III, (**C**) ANT of *S. niitakayamensis* with numerous small, protuberant secondary rhinaria on ANT III-VI (red arrows), (**D**) ANT VI of *M. sorini* with one of the accessory rhinaria moved to the PT, (**E**) ANT VI of *P. imbricatus* with accessory rhinaria in one group under the major rhinarium, (**F**) ANT VI of *S. niitakayamensis* with one of the accessory rhinaria moved to the PT (black arrow) and the accessory rhinaria near the major rhinarium and numerous secondary rhinaria, (**G**) uniformly brown pigmented fore wing of *M. sorini*, (**H**) only basally pigmented fore wing of *P. imbricatus*, and (**I**) unpigmented fore wing of *S. niitakayamensis*. The red dotted line shows major rhinaria, and the black dotted line shows accessory rhinaria arrangement; Pt—pterostigma, Rs—radial sector, M—media, and Cu_1a_ and Cu_1b_—cubital veins.

The new genus is morphologically and biologically related to *Pyrolachnus* and *Sinolachnus* by some general characteristics. Among all species belonging to *Pyrolachnus*, *Miyalachnus* differs in some significant characteristics, such as:

1. It has a much smaller body size (the lengths never overlap) (see Figure 1);
2. One accessory rhinarium on ANT VI is separate from others and lies on the processus terminalis, always above the major rhinarium and the group of remaining accessory rhinaria (including in the alate viviparous females and larvae).
3. In contrast to other *Pyrolachnus* species, *Miyalachnus* is characterized by the dorsal side of the head, thorax, and abdomen being covered with clearly visible denticles (in *Pyrolachnus*, the dorsum is smooth, or at most, imbricated but never with denticles). These features, as well as the smaller body size, make *Miyalachnus* close to *Sinolachnus*, but it differs from this genus in the greater number of sense pegs on the first segment of the fore tarsi—13–21 (7–9 in *Sinolachnus* and only 4–5 in *Pyrolachnus*)—and the dorsum of the head with denticles (in *Sinolachnus*, the head is smooth). Alate viviparous females in *Pyrolachnus* are characterized by colorless fore wings with only the basal part being dusky.
4. The fore wings of *Miyalachnus* are uniformly brown-pigmented. *Miyalachnus* alate morphs are characterized by pterostigma with a blunt distal end, in contrast to the pointed end in the representatives of *Sinolachnus*. Alate viviparous females of both genera differ greatly in ANT secondary rhinaria, which, in *Miyalachnus*, are big, rounded, and few in number (no more than 11), distributed only on ANT III and IV, while in *Sinolachnus*, all ANT segments bear a large number (several dozen to over 100) of very small, protuberant secondary rhinaria (see Figure 2). Detailed morphological and morphometric differences between *Pyrolachnus*, *Sinolachnus*, and *Miyalachnus* are given in Table 1.

***Description***

*Apterous viviparous female*. The body is medium to small, pear-shaped, densely covered with numerous long, fine, pointed setae. The head, pronotum, and mesonotum are uniformly sclerotized. The head has triommatidia on poorly developed ocular tubercles that are almost fused with the eyes. The frons is very slightly semi-circular, slightly concave in the middle, with a distinct epicranial suture. ANT 6-segmented has secondary rhinaria on ANT III–IV. ANT I is as long as or shorter than ANT II, which is narrower at the base and rounded at the apex. ANT III is shorter than ANT IV + V+VI and much narrower at the base than at the apex. The basal part of ANT III has 2–4 imbrications in the form of rounded rings. ANT IV is shorter than ANT V. ANT V is shorter than ANT VI with one big, rounded primary rhinarium without a sclerotic rosette on the expanded apex. ANT VI has a thin PT with one extra accessory rhinarium on the basal part of the PT over the major rhinarium and other accessory rhinaria. It has major rhinaria without sclerotic rosette. ANT III and IV sometimes have very small, rounded secondary rhinaria at the apex. ANT VI has 15–22 basal, 5 apical, and 2 subapical setae. It has a mesosternum with well-developed, sessile furca. The arms of the mesosternal furca have a broad median and strictured and hemispherical apical part. The legs are densely covered with numerous, very fine, and pointed setae, as long as or only slightly longer than the width of the hind tibiae. The first tarsal segments of the fore and middle legs have large numbers of peg-like setae. The abdomen has a membranous cuticle, with clearly visible denticles. ABD VIII has a dorsal cross-band, often with a gap in the spinal area. It has SIPH on large, rounded, or slightly oval cones with denticles and small pores with a poorly developed flange, densely covered with very fine, pointed setae. The subgenital plate is very big. The cauda is circular or semi-circular, with 20–25 long, pointed setae, which are longer and slightly thicker than those on the abdomen. The anal plate is well-developed and sclerotized.

*Alate viviparous female*. The fore wings of alate viviparous females are uniformly brown-pigmented on the whole surface of the wing membrane. The fore wings are imbricated with typical venation; the pterostigma is about 5–6 times longer than the width, but not more than 0.25 the length of Rs with a slightly pointed end. Rs is slightly curved at the base and then runs straight to the tip of the wing. The media are twice-branched, not reaching the edges of the wing. The hind wings have two oblique veins. The abdomen has a membranous cuticle, slightly imbricated with denticles. The abdomen is membranous and densely covered with long, fine, pointed setae and with dark intersegmental muscle sclerites and marginal plates on ABD I–III. The SIPH, cauda, genital, and anal plates are brown.

*First instar larvae*. In the mounted specimens, the head is sclerotized, light brown to brown. ANT 5-segmented, with PT longer than the BASE. Meso- and metathorax with marginal sclerotic plates (wings buds). The rostrum is long, reaching the SIPH. The abdomen is membranous with intersegmental muscle sclerites. The SIPH sclerites are very small, in the form of irregular rings around the pores. The body is densely covered with long, fine, pointed setae.

***Etymology***

We have great pleasure in naming the new genus to honor our dear colleague and friend Masahisa Miyazaki, a prominent Japanese aphidologist from the Inventory Center, National Institute for Agro-Environmental Sciences, Kannondai, Tsukuba, Ibaraki, who also fully supported our studies in this field and let us call him “Miya”.

***Gender***: masculine.

 

**Keys to all known morphs of species of the genus *Miyalachnus***

*Key to fundatrices:*

1. Appendages uniformly yellow to brown. URS with 15 accessory setae, LS/BD III 2.28–2.83. ABD II-IV without sclerites or scleroites … ***M***. ***nipponicus***

**-**  Appendages with darker distal parts. URS with 9–10 accessory setae, LS/BD III 1.62–2.00. ABD II-IV with sclerites and scleroites … ***M***. ***sorini* sp**. **nov**.

 

*Key to apterous viviparous females:*

1. Body poorly sclerotized, at most in the form of scattered scleroites and sclerites on ABD I and the spinal part of ABD VI and VII. URS with 15–18 accessory setae. The appendages are uniformly yellow or light brown. PT/BASE 0.90–0.96. URS/HT II 0.62–0.68 … ***M***. ***nipponicus***

**-**  Body strongly sclerotized, abdomen with sclerotized cross-bars or a solid sclerotic shield. URS with 9–10 accessory setae. Appendages with darker apical parts. PT/BASE 0.73–0.84. URS/HT II 0.58–0.60 … ***M***. ***sorini* sp**. **nov**.

 

*Key to alate viviparous females:*

1. PT/BASE 0.40–0.66. URS/ANT VI 0.75–0.83. URS with 15–18 accessory setae. FT I with 10–11 sense pegs. The common node of M1 and M2 under the rise of radial sector … ***M***. ***nipponicus***

**-**  PT/BASE 0.70–0.71. URS/ANT VI 0.64–0.71. URS with 10–11 accessory setae. FT I with 18–10 sense pegs. The common node of M1 and M2 under the tip of pterostigma … ***M***. ***sorini* sp**. **nov**.

 

*Key to first instar larvae:*

1. Appendages are uniformly yellow to pale brown. HW/ANT 0.85–0.90. PT/BASE 1.38–1.50 … ***M***. ***nipponicus***

**-**  Appendages with darker distal parts. HW/ANT 0.79–0.80. PT/BASE 1.21–1.26 … ***M***. ***sorini* sp**. **nov**.

 

*Miyalachnus nipponicus* (Sorin, 2011) **comb**. **nov**.

=*Pyrolachnus imbricatus nipponicus* Sorin, 2011: 66

 

*Material examined*: **Paratypes**. JAPAN, Matsushiro, Nagano-ken, 15 September 1992, *Prunus yeodensis*, N. Hirukawa leg., three apt, no 41, NIAES; five apt, no 45, NIAES; five apt, no 49, NIAES; three apt and two larvae, 50, NIAES; 18 June 1993; *Prunus isakura ascendens* (=*Cerasus spachiana* f. *ascendens*), one al, one alatoid nymph, NIAES; **Other material**: JAPAN, Morioka, Iwate Pref., 13 May 1972, *Prunus yedoensis*, M. Miyazaki leg., two Fx, 7206, NIAES.

 

Fundatrix—description (n = 2)

**Figure 3:**
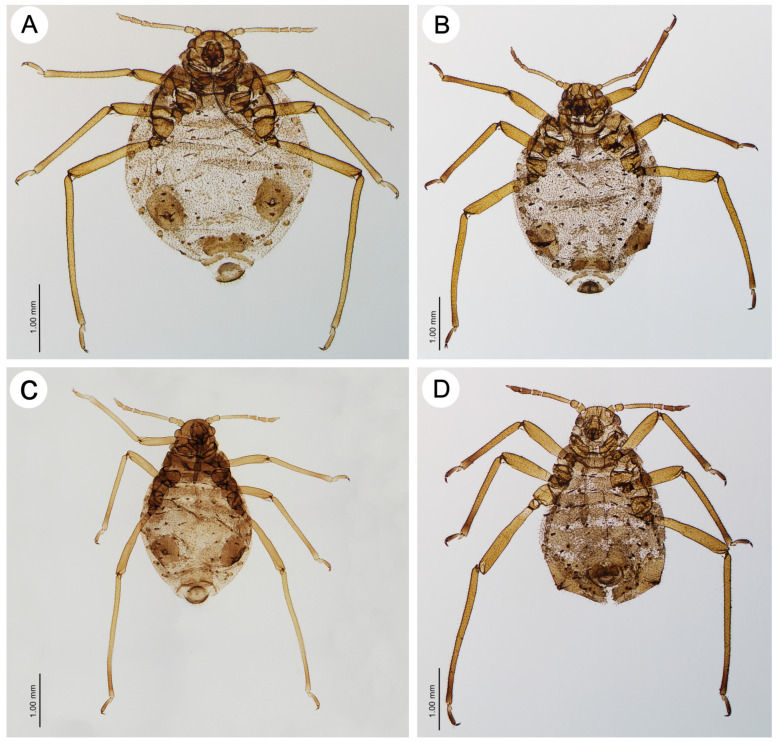
Apterous viviparous morphs of the genus *Miyalachnus*: (**A**) fundatrix of *M. nipponicus*, (**B**) fundatrix of *M. sorini*, (**C**) apterous viviparous female of *M. nipponicus*, and (**D**) apterous viviparous female of *M. sorini*.

*Colour*. In life: unknown. Pigmentation of mounted specimens: head and thorax sclerotized, light brown. ANT is uniformly yellow to light brown. The legs are uniformly yellow to light brown. The abdomen has light brown sclerotization. The SIPH, cauda, and subgenital and anal plates are light brown (Figure 3A). *Morphometric characters*: HW 0.57–0.58 × ANT. Head setae, 0.070–0.090 mm long. ANT is about 0.34 × BL. ANT III and ANT IV have one secondary rhinarium. PT 0.53–0.57 × BASE. Other antennal ratios: ANT VI:ANT III 0.41–0.46; ANT V:ANT III 0.28–0.32; ANT IV:ANT III 0.26–028. ANT setae 0.060–0.085 mm long. LS 2.28–2.83 × BD III. URS 0.32–0.34 × ANT III, 0.73–0.78 × ANT VI, and about 0.53 × HT II, with 15 accessory setae. Hind legs 1.06–1.08 × BL with fine, pointed setae, 0.060–0.075 mm long. FT I with 7–8, MT I with 4–5, and HT I with 2 sense pegs. HT II 0.60–0.65 × ANT III and 1.39–1.46 × ANT VI. The abdomen is membranous, with small, spino-pleural areas of scleroites on ABD I and the spinal plates of big, brown, spinal scleroites (sometimes fused into larger sclerites) on ABD I and V–VI, two large sclerotic plates on ABD VII, and a broken cross-bar on ABD VIII. The dorsal abdominal setae are 0.07−0.100 mm long. Measurements are given in Table 2.

*Remarks*: This so far unknown morph differs from the apterous viviparous female in many characteristics, such as body shape and size, a lower ratio of ANT/BL, a lower ratio of PT/BASE, and fewer sense pegs on FT I.

 

Apterous viviparous female—redescription (n = 11)

**Figure 4:**
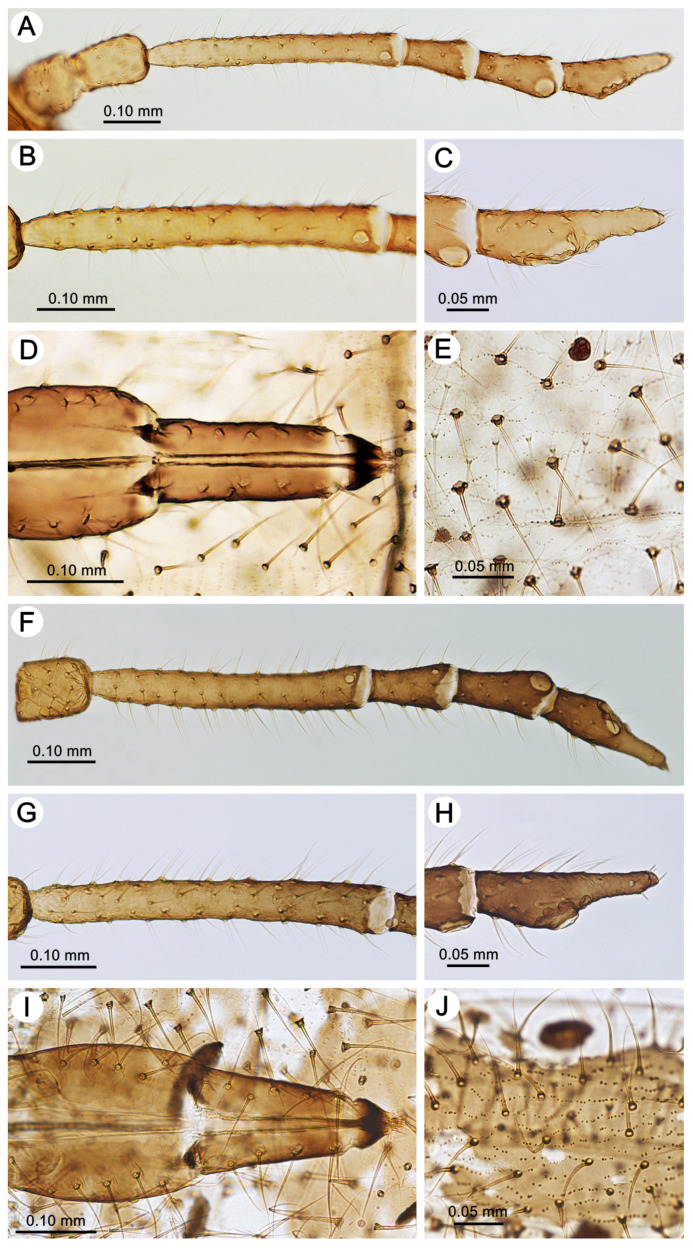
Characters of apterous viviparous females of the genus *Miyalachnus*: (**A**) ANT, (**B**) ANT III, (**C**) ANT VI, (**D**) URS, (**E**) abdominal membranous cuticle of *M. nipponicus*, (**F**) ANT, (**G**) ANT III, (**H**) ANT VI, (**I**) URS, and (**J**) abdominal sclerotized cuticle of *M. sorini*.

**Figure 5:**
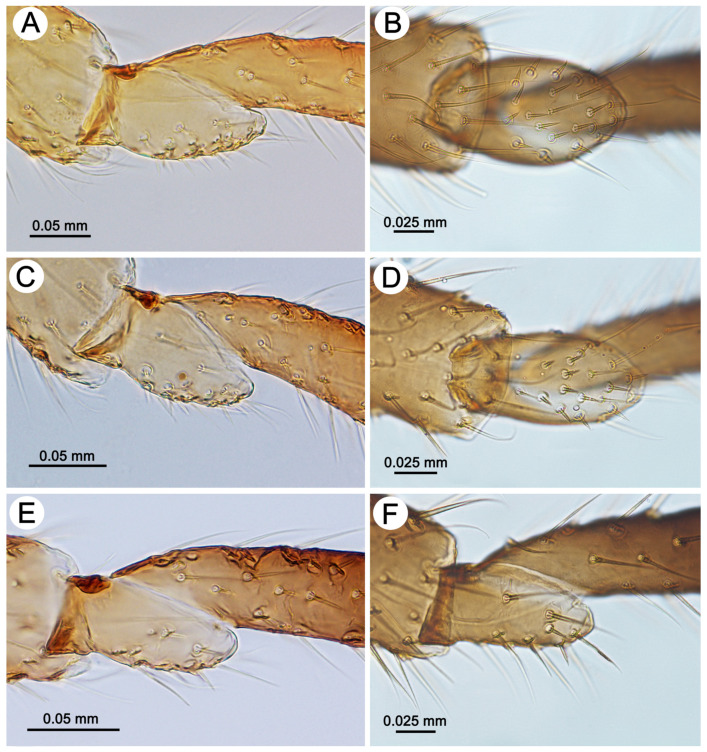
First segment of tarsi sense pegs of apterous viviparous females of the genus *Miyalachnus*: (**A**) FT I of *M. nipponicus*, (**B**) FT I of *M. sorini*, (**C**) MT I of *M. nipponicus*, (**D**) MT I of *M. sorini*, I (**E**) HT I of *M. nipponicus*, and (**F**) HT I of *M. sorini*.

*Colour*. In life: unknown. Pigmentation of mounted specimens: head and thorax sclerotized, brown. ANT is uniformly yellowish-brown or light brown. The legs are uniformly yellowish-brown or light brown. The abdomen is membranous, with brown sclerotization. The SIPH cones are brown. The cauda and the subgenital and anal plates are brown (Figure 3C). *Morphometric characters*: HW 0.53–0.60 × ANT. Head setae, 0.060–0.105 mm long. ANT (Figure 4A), 0.40–0.42 × BL. ANT III with 0–1 (Figure 4B), and ANT IV has 0–2 secondary rhinaria. PT 0.90–0.96 × BASE (Figure 4C). Other antennal ratios: ANT VI:ANT III 0.45–0.55; ANT V:ANT III 0.31–0.36; ANT IV:ANT III 0.23–0.31. ANT setae 0.060–0.090 mm long. LS 2.66–3.20 × BD III. URS (Figure 4D) is narrow on the whole length, with a pointed apical part, 0.37–0.43 × ANT III, 0.77–0.88 × ANT VI, and 0.62–0.68 × HT II, with 15–18 accessory setae. Hind legs 1.05–1.17 × BL with fine, pointed setae, 0.055–0.085 mm long. FT I (Figure 5A) with 13–16, MT I (Figure 5C) with 8–12, and HT I (Figure 5E) with 2–3 sense pegs. HT II 0.54–0.62 × ANT III and 1.17–1.30 × ANT VI. The dorsal side of the abdomen is membranous, with poorly developed sclerotization (Figure 4E), mostly in the form of small scleroites (on ABD I and II on the whole surface and sometimes fused in larger ones) (Figure 3C). The dorsal abdominal setae are 0.080–0.110 mm long. The genital plate is in the form of a single sclerite. The measurements are given in Table 2.

Alate viviparous female—redescription (n = 1)

**Figure 6:**
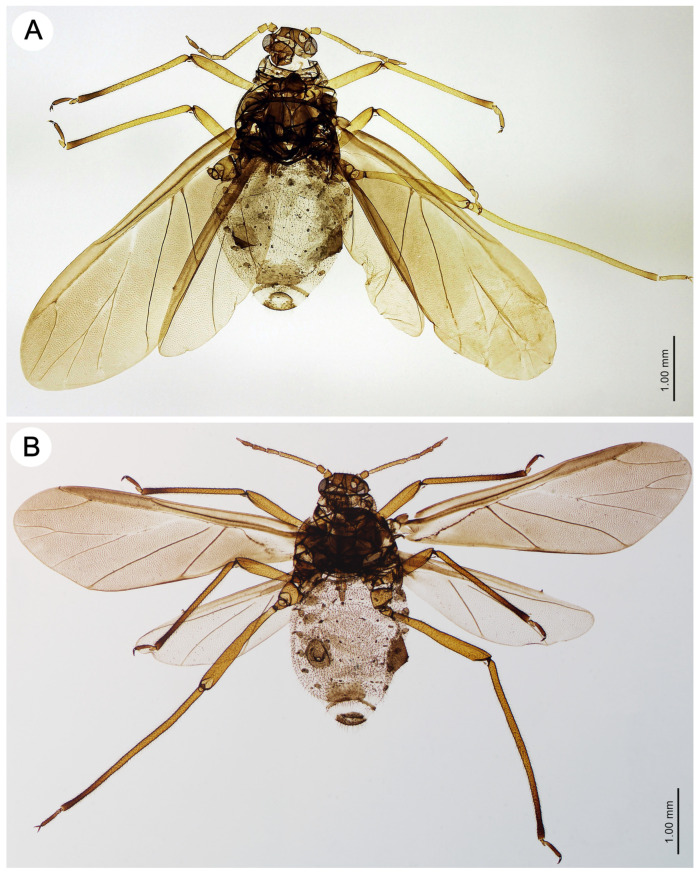
Alate viviparous females of the genus *Miyalachnus* of (**A**) *M. nipponicus* and (**B**) *M. sorini*.

**Figure 7:**
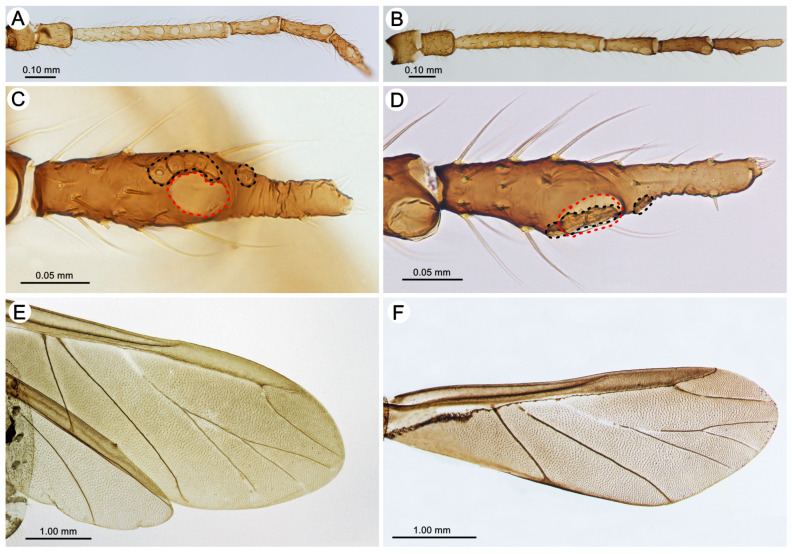
Characters of alate viviparous females of the genus *Miyalachnus*: (**A**) ANT of *M. nipponicus*, (**B**) ANT of *M. sorini*, (**C**) ANT VI of *M. nipponicus*, (**D**) ANT VI of *M. sorini*, (**E**) fore wing of *M. nipponicus*, and (**F**) fore wing of *M. sorini*, red dotted line indicates major rhinarium, black dotted line indicates accessory rhinaria.

*Colour*. In life: unknown. Pigmentation of mounted specimens: head and pronotum sclerotized, light brown to brown. The rest of the thorax is sclerotized and dark brown. ANT is light brown with ANT V and VI brown. The legs are yellow, with brown distal parts of the tibiae and HT II (Figure 6A). *Morphometric characters*: HW 0.48–0.50 × ANT. Head setae, 0.100–0.110 mm long. ANT (Figure 7A), 0.38–0.42 × BL. ANT III with 8–10, ANT IV with 1–2, and ANT V, sometimes with 1–2 secondary rhinaria. PT 0.40–0.66 × BASE (Figure 7C). Other antennal ratios: ANT VI:ANT III 0.40–0.43; ANT V:ANT III 0.33–0.34; ANT IV:ANT III 0.30–0.33. ANT setae 0.080–0.090 mm long. LS 2.25–2.42 × BD III. URS 0.30–0.36 × ANT III, 0.75–0.83 × ANT VI, and 0.54–0.55 × HT II, with 15–16 accessory setae. The setae on the hind legs are fine and pointed and 0.095–0.110 mm long. FT I with 10–11, MT I with 11–12, and HT I with 3 sense pegs. HT II 0.56–0.58 × ANT III and 1.39–1.44 × ANT VI. The common node of M1 and M2 starts under the rise of the radial sector (Figure 7E). The dorsal abdominal setae are 0.100–0.110 mm long. The measurements are given in Table 2.

First instar larva—description (n = 2)

(Figure 8A)

HW 0.85–0.90 × ANT. Head setae, 0.035–0.085 mm long. ANT 0.38–0.46 × BL. PT 1.38–1.50 × BASE. Other antennal ratios: ANT V:ANT III 0.88–0.91; ANT IV:ANT III about 0.58. ANT setae 0.050–0.070 mm long. LS 1.71–1.75 × BD III. URS 0.94–0.97 × ANT III, 1.03–1.10 × ANT VI, and 0.76–0.82 × HT II with 7–9 accessory setae. Hind legs 1.80–2.41. The setae on the hind legs are 0.035–0.060 mm long. FT I with 2–4, MT I with 3, and HT I with 1–2 sense pegs. HT II 1.17–1.23 × ANT III and 1.33–1.35 × ANT VI. The measurements are given in Table 2.

*Diagnosis*. *Miyalachnus nipponicus* can be easily recognized from the new species by the following characteristics: (**1**) poorly sclerotized body, at most in the form of scattered scleroites and sclerites on ABD I and the spinal part of ABD VI and VII (the body is strongly sclerotized in *M. sorini*); (**2**) URS with 15–18 accessory setae (9–10 in *M. sorini*); (**3**) uniformly yellow or light brown appendages (appendages dark brown in *M. sorini*); (**4**) PT/BASE 0.90–0.96 (PT/BASE 0.73–0.84 in *M. sorini*). Detailed differences between known morphs of *M. nipponicus* and *M. sorini* are given in Table 3

*Distribution*. Matsushiro, Nagano Prefecture, Honsû Island, Japan.

*Biology*. The species lives in colonies on the twigs of *Prunus yedoensis* (Sorin, 2011). One nymph and alate viviparous female were also found on *Cerasus spachiana* f. *ascendens* (=*Prunus spachiana*) (data from the slide, not mentioned in the description). Apterous viviparous females, alatoid nymphs, and alate viviparous females were collected in July, and apterous viviparous females and larvae were collected in September. There is no information about ant attendance.

 

*Miyalachnus sorini* **sp**. **nov**.

 

*Material examined*. **Holotype**. Japan, Uschiuku, Ibaraki Pref., 19 May 2016, *Prunus buergeriana*, M. Miyazaki leg., one apt, J/05/16/1, DZUS; Paratypes. Kessoku, 18 Apr 2017, *P*. *buergeriana*, M. Miyazaki leg., two first instar larvae, J/04/17/5, DZUS; Rokuto, Ibaraki, Pref., *Cerasus jamasakura*, three first instar larvae, J/04/17/6; one Fx, J/04/17/4, DZUS; two Fx, J/04/17/2; Kessoku, *P*. *buergeriana*, one fundatrix, J/04/17/1, NIAES; two fx, J/04/17/2, DZUS; Tsukuba, 8 June 2016, *C*. *jamasakura*, three apt J/06/16/1, DZUS; three apt, J/06/16/2, NHMUK; Uschiuku, 19 May 2016, *P*. *buergeriana*, two apt, J/05/16/2, NIAES; four al, J/05/16/4, DZUS, three al, J/05/16/3, NIAES; Rokuto, 21 October 2016, *Cerasus lannesiana*, four apt, J/10/16/1, DZUS; three apt, J/10/16/2, DZUS; 22 October 2016, two ♀, J/11/16/1, DZUS, two ♀, J/11/16/2, NIAES, three ♀, J/11/16/3, DZUS; one ♂, J/11/16/4, DZUS; one ♂, J/11/16/5, DZUS.

 

Fundatrix—description (n = 6)

*Colour*. In life: head and thorax black, ANT dark brown. The abdomen is grey, with black markings in the form of paired rectangle spots, which are larger in the front and smaller in the back of the abdomen. The SIPH and cauda are black. The legs are brown, with darker distal parts of tibiae and tarsi. The larvae of the fundatrices are dark brown (see the biology section). Pigmentation of mounted specimens: head and thorax sclerotized, yellowish-brown. ANT is yellow, with ANT V and VI (sometimes apical part of ANT IV) being brown. The legs are yellow, with darker distal parts of the tibiae and HT II. The abdomen has brown sclerotization. The SIPH cones, cauda, and subgenital and anal plates are brown (Figure 3B). *Morphometric characters*: HW 0.53–0.60 × ANT. Head setae, 0.060–0.100 mm long. ANT 0.30–0.37 × BL. ANT III and ANT IV with 0–1 secondary rhinaria. PT 0.50–0.56 × BASE. Other antennal ratios: ANT VI:ANT III 0.41–0.46; ANT V:ANT III 0.29–0.31; ANT IV:ANT III about 0.25. ANT setae 0.050–0.080 mm long. LS 1.62–2.00 × BD III. Rostrum reaching ABD I-III. URS 0.34–0.35 × ANT III, 0.76–0.83 × ANT VI and about 0.58 × HT II, with 9–10 accessory setae. Hind legs 0.87–1.06 × BL with fine and pointed setae, 0.050–0.070 mm long. FT I with 7–8, MT I with 6–7 and HT I with 1–2 sense pegs. HT II 0.58–0.60 × ANT III and 1.31–1.41 × ANT VI. The abdomen is membranous, with large, brown, spino-pleural, ragged sclerites on ABD I and spinal plates of large, brown, spinal scleroites (sometimes fused into larger sclerites) on ABD II–VI, two large sclerotic plates on ABD VII, and a broken cross-bar on ABD VIII. The dorsal abdominal setae are 0.075–0.100 mm long. The measurements are given in Table 4.

*Remarks*: This morph differs from the apterous viviparous female in many characteristics, including the body shape and pigmentation of live specimens. In mounted slides, the fundatrix differs from apterous viviparous females in having a bigger body, abdomen sclerotization, higher ratio of PT/BASE, shorter antennal setae, and fewer sense pegs on FT I.

 

Apterous viviparous female—description (n = 18)

**Figure 9:**
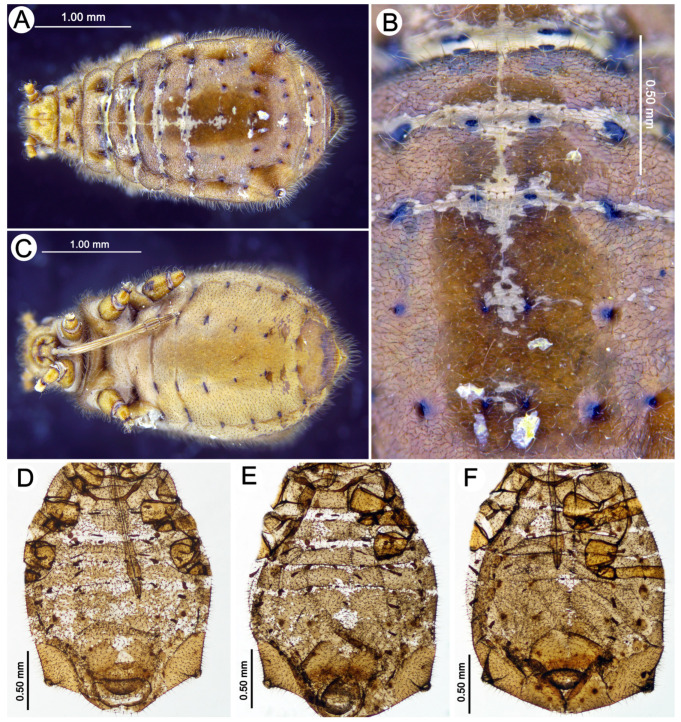
Body sclerotization of apterous viviparous female of *M. sorini*: (**A**) general view of the sclerotized dorsal side of the body, (**B**) dorsal abdominal sclerotic shield with visible denticles and a poorly developed membranous area, (**C**) ventral side of the body with pleural sclerotic plates on ABD sternite VI, and (**D**–**F**) different degrees of abdominal sclerotization from fused sclerites and scleroites to solid sclerotic shield.

*Colour*. In life: head and pronotum brown, ANT brown with darker distal segments. The rest of the thorax and abdomen mat is black (in freshly mounted individuals shiny), with greyish, oval spots in the spinal position and pleuro-marginal spots on meso- and metanotum and on ABD IV near the bases of SIPH legs from yellow-orange to red-brown. The larvae and nymphs are orange-red-brown (see the biology section). Pigmentation of mounted specimens: head, thorax and abdomen sclerotized, yellowish-brown. ANT is yellowish-brown, with ANT V and VI (sometimes IV-VI) being brown. The legs are yellowish-brown, with brown distal parts of the tibiae and HT II. The abdomen is sclerotized, with large, brown lateral stripes and dark intersegmental muscle sclerites. The SIPH cones are light brown. The cauda and the subgenital and anal plates are brown (Figure 3D). *Morphometric characters*: HW 0.54–0.60 × ANT. Head setae, 0.080–0.110 mm long. ANT (Figure 4F), 0.39–0.43 × BL. ANT III (Figure 4G) and ANT IV with 0–1 secondary rhinaria. PT 0.73–0.84 × BASE (Figure 4H). Other antennal ratios: ANT VI:ANT III 0.46–0.53; ANT V:ANT III 0.34–0.36; ANT IV:ANT III 0.28–0.34. ANT setae 0.065–0.100 mm long. LS 2.42–2.85 × BD III. URS (Figure 4I) is wider at the base than at the apex with a pointed apical part, 0.35–0.40 × ANT III, 0.69–0.78 × ANT VI and 0.58–0.60 × HT II, with 10–11 accessory setae. Hind legs 1.23–1.34 × BL with fine, pointed setae, 0.075–0.085 mm long. FT I (Figure 5B) with 18–21, MT I (Figure 5D) with 13–14, and HT I (Figure 5F) with 2 sense pegs. HT II 0.61–0.68 × ANT III and 1.15–1.31 × ANT VI. The dorsal side of the abdomen is strongly sclerotized, with poorly developed membranous areas between segments or as small spinal patches (Figure 9A,B). The ventral side of the abdomen is membranous with sclerites and scleroites on ABD sternites VI and VII (Figure 9C). Abdominal sclerotization varies from lateral stripes of sclerites and scleroites with more membranous areas and solid cross bars to a total sclerotic shield (Figure 9D–F). The cuticular denticles are well-developed (Figure 4J). The dorsal abdominal setae are 0.090–0.110 mm long. The genital plate is in the form of two separate sclerites. The measurements are given in Table 4.

*Remarks*. Autumnal apterous viviparous females are more similar to oviparous females in reduced dorsal abdominal sclerotization.

Alate viviparous female—description (n = 7)

*Colour*. In life: head and pronotum dark brown to black, ANT brown with darker distal segments. The rest of the thorax is dark grey, and the abdomen mat black has greyish, oval spots in the spinal position and pleuro-marginal spots on ABD IV near the bases of SIPH, which are darker than the rest of the abdomen. The wings are black. The legs are red-brown to brown. In freshly mounted individuals, the body is shiny and brown, with white wings and yellow legs. The alatoid nymphs are red-brown. The same pigmentation characterizes the last instar nymphs and adult alate (see the biology section). Pigmentation of mounted specimens: head and thorax sclerotized, brown to dark brown. ANT is brown, with ANT V and VI (sometimes apical part of ANT IV) being brown. The legs are yellowish-brown, with brown distal parts of tibiae and HT II (Figure 6B). *Morphometric characters*: HW 0.49–0.52 × ANT. Head setae, 0.100–0.120 mm long. ANT (Figure 7B), 0.36–0.40 × BL. ANT III with 7–11, ANT IV with 1–3 and ANT V sometimes with 0–1 secondary rhinaria. PT 0.70–0.76 × BASE (Figure 7D). Other antennal ratios: ANT VI:ANT III 0.45–0.48; ANT V:ANT III 0.33–0.36; ANT IV:ANT III 0.31–0.35. ANT setae 0.080–0.100 mm long. LS 2.28–2.85 × BD III. URS 0.31–0.32 × ANT III, 0.64–0.71 × ANT VI and 0.51–0.57 × HT II, with 10–11 accessory setae. The setae on the hind legs are fine, pointed, and 0.080–0.100 mm long. FT I with 18–20, MT I with 14–15 and HT I with 2 sense pegs (see the SEM description). HT II 0.56–0.62 × ANT III and 1.21–1.32 × ANT VI. The common node of M1 and M2 starts under the tip of the pterostigma (Figure 7F). The dorsal abdominal setae are 0.090–0.120 mm long. The measurements are given in Table 4.

 

Apterous male—description (n = 2)

**Figure 10:**
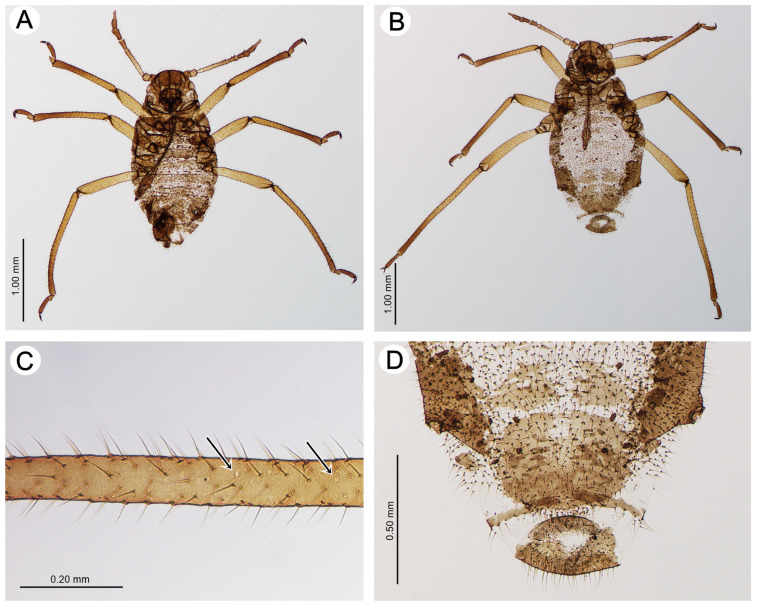
Sexual morphs of *M. sorini*: (**A**) apterous male, (**B**) oviparous female, (**C**) poorly visible scent plaques on hind tibiae of oviparous female (black arrows), and (**D**) large genital plate on the oviparous female abdomen.

*Colour*. In life: body dark brown with lighter appendages. The abdomen has poorly visible small grey spinal patches on ABD I–VI and pleural ones on AND I and IV (see the biology section). Pigmentation of mounted specimens: head and thorax sclerotized, brown. ANT is brown, with a pale brown ANT III. The legs are light brown, with brown distal parts of the tibiae and HT II. The abdomen has brown sclerotization. The SIPH cones, cauda, anal plate, and genitalia are brown (Figure 10A). *Morphometric characters*: HW 0.54–0.60 × ANT. Head setae, 0.060–0.080 mm long. ANT 0.54–0.60 × BL. ANT III with 0–3, ANT IV with 0–1 secondary rhinaria. PT 0.90–1.14 × BASE. Other antennal ratios: ANT VI:ANT III 0.59–0.63; ANT V:ANT III about 0.36; ANT IV:ANT III 0.30–0.31. ANT setae 0.045–0.070 mm long. LS about 2.80 × BD III. URS 0.44–0.50 × ANT III, 0.75–0.78 × ANT VI and about 0.68 × HT II, with 10 accessory setae. Hind tibiae with setae, 0.050–0.070 mm long. FT I with 14–15, MT I with 8–9 and HT I with 1–2 sense pegs. HT II 0.65–0.72 × ANT III and 1.11–1.14 × ANT VI. The dorsal side of the abdomen is membranous, with ragged cross-bands on all segments. The dorsal abdominal setae are 0.060–0.080 mm long. The genitalia are sclerotized. The measurements are given in Table 4.

Oviparous female—description (n = 7)

*Colour*. In life: head and pronotum dark brown, ANT brown with darker distal segments. The rest of the thorax and abdomen are dark brown to black. Greyish spots, if present, are only in a spinal position on ABD IV and/or ABD V and in a marginal position on ABD V near SIPH bases. The legs are brown, with darker distal parts (see the biology section). Pigmentation of mounted specimens: head and thorax sclerotized, brown. ANT is yellow to pale brown, with ANT IV–VI being brown. The legs are light brown, with brown distal parts of tibiae and HT II. The abdomen has brown sclerotization. The SIPH cones, cauda, and subgenital and anal plates are brown (Figure 10B). *Morphometric characters*: HW 0.54–0.58 × ANT. Head setae, 0.080–0.085 mm long. ANT 0.37–0.39 × BL. ANT III and IV with 0–1 secondary rhinaria. PT 0.69–0.83 × BASE. Other antennal ratios: ANT VI:ANT III 0.44–0.57; ANT V:ANT III 0.32–0.36; ANT IV:ANT III 0.28–0.29. ANT setae 0.040–0.085 mm long. LS 2.12–2.50 × BD III. URS 0.32–0.42 × ANT III, 0.72–0.79 × ANT VI and 0.60–0.66 × HT II, with 10–12 accessory setae. The hind tibiae are not swollen, with fine, pointed setae, 0.050–0.080 mm long and 10–25 small, rounded or irregular, poorly visible pseudosensoria located from the proximal part to about half of their length (Figure 10C). FT I with 12–14, MT I with 7–8 and HT I with 2 sense pegs. HT II 0.53–0.65 × ANT III and 1.13–1.21 × ANT VI. The dorsal side of the abdomen is membranous, with spial areas of sclerites or scleroites on ABD I–VIII, a broken cross-bar on ABD VIII, and spinal plates or sclerites on ABD I–IV. The dorsal abdominal setae are 0.075–0.120 mm long. The ventral side has large sclerotic plates on ABD sternite V and a large, slightly oval genital plate with an indentation on the proximal end (Figure 10D). The measurements are given in Table 4.

 

First instar larva—description (n = 5)

(Figure 8B)

HW 0.79–0.80 × ANT. Head setae, 0.065–0.090 mm long. ANT 0.42–0.44 × BL. PT 1.21–1.26 × BASE. Other antennal ratios: ANT V:ANT III 0.91–0.94; ANT IV:ANT III 0.55–0.64. ANT setae 0.045–0.055 mm long. LS 1.10–1.37 × BD III. URS 0.94–1.00 × ANT III, 1.00–1.09 × ANT VI and 0.77–0.80 × HT II, with 5–7 accessory setae. Hind legs 0.81–0.92 × BL. Setae on hind legs 0.040–0.075 mm long. FT I with 4–6, MT I with 3 and HT I with 1–2 sense pegs. HT II 1.16–1.29 × ANT III and 1.23–1.41 × ANT VI. The measurements are given in Table 4.

 

*Diagnosis*. Apterous viviparous females of the new species may be easily distinguished from *M*. *nipponicus* by (**1**) strongly sclerotized abdomen (membranous in *M*. *nipponicus*), (**2**) darker parts of appendages (uniformly pigmented in *M*. *nipponicus*), and (**3**) lower ratio of PT/BASE 0.73–0.84 (0.90–0.96 in *M*. *nipponicus*). Alate viviparous females are characterized by differences in wing venation: (**1**) common node of M1 and M2 and point of radial sector rising, (**2**) higher ratio of PT/BASE, 0.70–0.71 (0.40–0.66 in *M*. *nipponicus*), and (**3**) larger number and more sense pegs on FT I, 18–20 (10–11 in *M*. *nipponicus*). Moreover, all morphs of both species differ in the number of accessory setae on URS. Detailed differences between all known morphs of *M*. *nipponicus* and *M*. *sorini* are given in Table 3.

*Etymology*. The authors have the pleasure of naming the new species in honor of a prominent aphid specialist, the late Masato Sorin from Kogakkan University, Kuratayama, Ise, Mie, who also worked on Lachninae aphids for several years and described the first species from the new genus.

*Distribution*. Numerous samples of the new species have been collected in Ibaraki Prefecture in Japan, mainly in Uschiuku, Kessoku, Rokuto, and Tsukuba.

### 3.2. Miyalachnus Morphology and Sensilla (Based on the Type Species M. sorini)

#### 3.2.1. General Morphology

**Figure 11:**
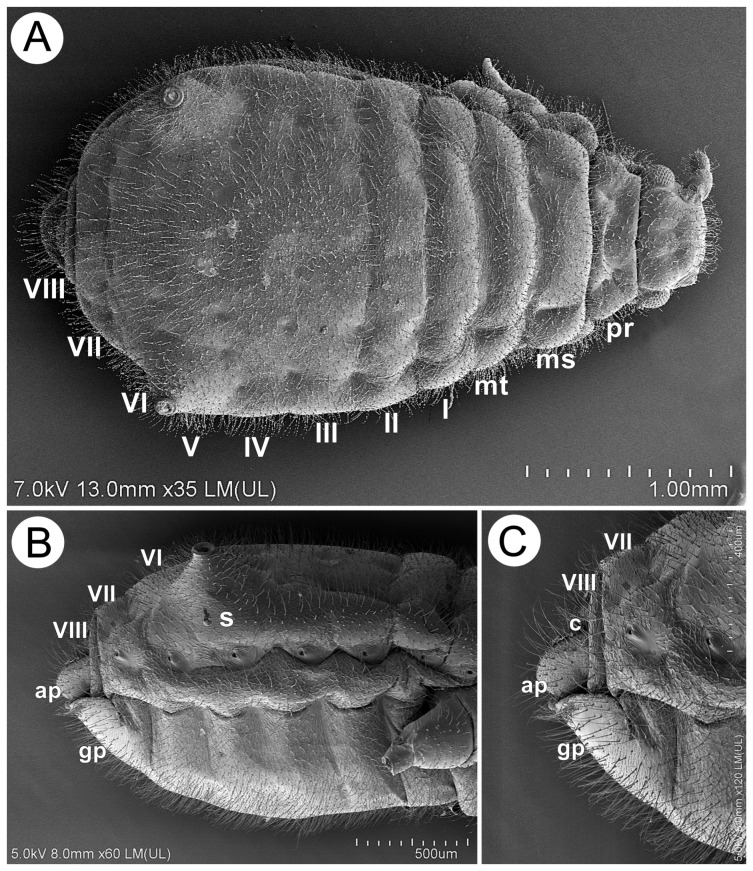
Scanning electron microscopy of an apterous viviparous female of *M. sorini*: (**A**) dorsal side of the body and segmentation, (**B**) lateral side of the abdomen, and (**C**) lateral side of the end of the abdomen: pr—pronotum, ms—mesonotum, mt—metanotum, I–VIII—abdominal tergites I–VIII, s—SIPH, c—cauda, ap—anal plate, and gp—genital plate.

Apterous viviparous females of *Miyalachnus* are pear-shaped, with the body widest across ABD IV and V, and densely covered with numerous, very fine setae. The head is well-developed and separated from the pronotum, which is also separated from the mesonotum. The mesonotum and metanotum are not well separated, only with well-developed borders. On the abdomen, only ABD I and II are separated by borders, while ABD III–VI form a uniform shield with bordered ABD VII and VIII (Figure 11A). The abdominal part of the body is well separated into dorsal and ventral sides with a small lateral side with clearly visible spiracles (Figure 11B). The perianal structures are shortened, like in many myrmecophilous aphids. Abdominal tergite VIII is covered with ABD VII, the cauda is very short, and the anal plate is slightly raised, with long, fine setae (Figure 11C).

#### 3.2.2. Body Surface and Sensilla

**Figure 12:**
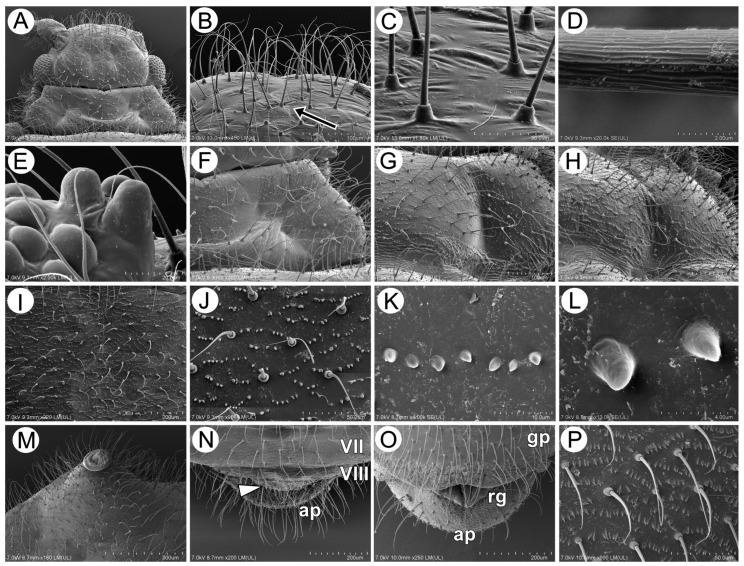
SEM of the head and dorsum of *M. sorini*: (**A**) head and pronotum structure, (**B**) frontal area with visible epicranial suture (arrow) and long setae (trichoid sensilla) with very fine, pointed apices, (**C**) fine structure of the head surface and trichoid sensilla sockets, (**D**) fine structure of trichoid sensillum, (**E**) fine structure of the ocular tubercle and triommatidium, (**F**) surface and chaetotaxy of pronotum, (**G**) surface and chaetotaxy of mesonotum, (**H**) surface and chaetotaxy of metanotum, (**I**,**J**) surface of cuticle and chaetotaxy of the abdomen, (**K**,**L**) fine structure of abdominal denticles, (**M**) structure and chaetotaxy of SIPH cone, (**N**) fine structure of the end of the dorsal abdomen, (**O**) fine structure of the end of the ventral abdomen, and (**P**) fine structure and chaetotaxy of the genital plate: VII, VIII—abdominal segments VII and VIII, arrowhead—cauda, ap—anal plate, gp—genital plate, and rg—rudimentary gonapophyses.

**Figure 13:**
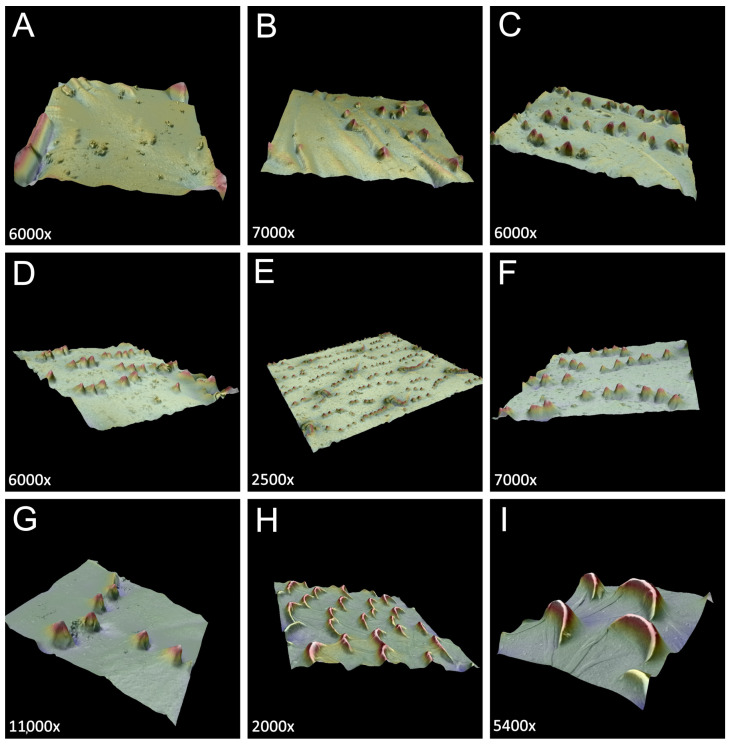
3D reconstruction of the body surfaces of *M. sorini*: (**A**) head surface, (**B**) pronotum surface, (**C**) mesonotum surface, (**D**) metanotum, (**E**–**G**) abdominal surface of an apterous viviparous female, and (**H**,**I**) surface of fore wings of an alate viviparous female.

The dorsal side of the body is densely covered with long, very fine, pointed setae—trichoid sensilla (Figure 12A). The surface of the head is smooth, with small wrinkles, and the high sockets of the trichoid sensilla are very clearly visible (Figure 12B,C). The sensilla surface is densely covered with many thin longitudinal, rib-like stripes (Figure 12D). The compound eyes are large, with clearly visible triommatidia on a well-separated ocular tubercle (Figure 12A,E). The pronotum, mesonotum, and metanotum are also densely covered with long, fine setae, and additionally, very small, numerous denticles are visible (fewer on the pronotum and more on the metanotum) (Figure 12F–H). A very similar situation may be noted on the surface of abdominal tergites. Their surface is covered with numerous, long, fine setae and a regular pattern of denticles in the form of transverse, slightly semi-circular rows (Figure 12I,J). The abdominal cuticle surface is completely smooth, and the denticles are in the form of very small cones with slightly curved apices (Figure 12K,L and Figure 13A–G). Siphunculi are in the form of regular, very hairy cones with a well-developed flange and operculum (Figure 12M). The dorsal side of the perianal structures shows that the cauda is very poorly visible and strongly pressed between ABD VIII and the anal plate (Figure 12N). The anal plate is big and rounded, with many very long, pointed setae (Figure 12O), and the genital plate is covered with small spicules (Figure 12P).

#### 3.2.3. Antennal Sensilla

**Figure 14:**
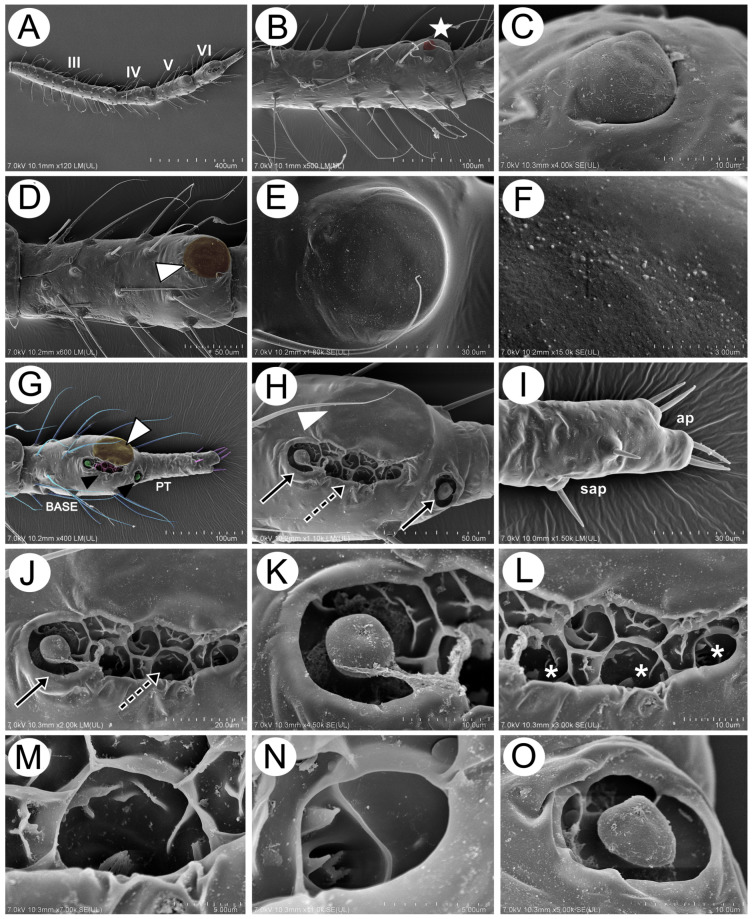
SEM of antennal characters and sensilla of apterous viviparous females of *M. sorini*: (**A**) structure of the ANT flagellum, (**B**) structure of the distal part of ANT III with long, hair-like trichoid sensilla and small multiporous placoid sensillum (star), (**C**) fine structure of the small multiporous placoid sensillum, (**D**) ANT V with large multiporous placoid sensillum, (**E**) fine structure of large multiporous placoid sensillum, (**F**) fine structure of the porous surface of the large multiporous sensillum, (**G**) ANT VI with accessory rhinaria (black arrowheads) separated into two groups, one on the PT and the rest near the major rhinarium (white arrowhead), (**H**) structure of the large placoid sensillum (white arrowhead), small placoid sensilla (solid arrows), and sunken coeloconic sensilla (dotted arrow), (**I**) trichoid sensilla of the distal part of PT divided into apical setae (ap) and subapical setae (sap), (**J**) structure of the accessory rhinaria cavity with small multiporous placoid sensillum (solid arrow) and sunken coeloconic sensilla (dotted arrow), (**K**) fine structure of the small placoid sensillum in the proximal position, (**L**) different types of sunken coeloconic sensilla (asterisks), (**M**,**N**) fine structure of two different types of sunken coeloconic sensilla, and (**O**) fine structure of the small placoid sensillum in the distal position.

The antennae in *Miyalachnus* are 6-segmented with long ANT III and rather short ANT IV–VI. The antennal segments carry many clearly visible, long setae which are type I trichoid sensilla (Figure 14A). The type I trichoid sensilla on the segments are very fine and more delicate from about one-third of their length and pointed. They lie in protuberant, semi-circular, or slightly trapezoidal sockets (Figure 14B,D). The antennal segments bear very few, small, multiporous placoid sensilla (secondary rhinaria), especially on the distal part of ANT III (Figure 14B). The small multiporous placoid sensillum on ANT III is slightly protuberant with a small cavity almost around it, about 14–16 μm in diameter and with about 20–23 pores per μm^2^ (Figure 14C). On the distal part of ANT V, a slightly protuberant, rounded, large multiporous placoid sensillum (primary rhinarium) is visible (Figure 14D). The sensillum is characterized by a very smooth edge, with a very poorly developed border between the edge and the porous membrane. There are no flanges or projections around the edge of the sensillum (Figure 14E). The surface of the porous membrane is smooth, and the pores are not clearly visible, about 15–20 per μm^2^ (Figure 14F). ANT VI is divided into an egg-shaped basal part (BASE) and a thin, finger-like terminal process (PT) with five kinds of sensilla (Figure 14G). The basal part of this segment is covered like other segments, with many long, fine, pointed type I trichoid sensilla and a group of sensilla on the distal part—the large multiporous placoid sensillum (major rhinarium), one small multiporous placoid sensillum, and sunken coeloconic sensilla (accessory rhinaria) (Figure 14G,H), which are in one group, immediately, next to the large multiporous placoid sensillum. As the basal part of the last antennal segment is determined by the end of the major rhinarium, the second accessory rhinarium—a small multiplacoid sensillum—is separate from other sensilla (placoid and coeloconic) and lies on the basal part of the PT (Figure 14H). The apical part of the PT of ANT VI is covered with seven type II trichoid sensilla. They are very short (about 10–15 μm long) and rigid, with slightly blunted apices, and lie in protuberant, semi-circular sockets. The type II trichoid sensilla form three separate groups: two groups (per three and two sensilla) on the very apical part of the segment (apical setae) and one group under them (subapical setae) (Figure 14I). As mentioned above, accessory rhinaria are separated from each other. The one large group is formed by one small placoid sensillum and sunken coeloconic sensilla. They all lie in a deep cavity with many transverse reinforcements, forming separate, open cells (Figure 14J). The first small placoid sensillum is rounded and mushroom-shaped, about 7.50–8.50 μm in diameter. The sensillum is surrounded by a wide, circular, and rolled-up flange (Figure 14K). The mentioned transverse reinforcements form four open cells, and in three of them, two kinds of sunken coeloconic sensilla are visible, with two more protruding (type II) sensilla and one more sunken (type I) sensillum, which is poorly visible (Figure 14L). Type II coeloconic sensilla are characterized by many (15–20) long, finger-like projections (Figure 14M), whereas type I coeloconic sensilla (the more sunken type) have only 6–8 shorter projections and lie deep in an additional, small circular cavity (Figure 14N). The separate small multiporous placoid sensillum, which lies on the basal part of the terminal process, is like the first one, i.e., mushroom-shaped and rounded, about 10–11 μm in diameter, surrounded by a small, protuberant, and inrolled flange with smooth edges (Figure 14O).

**Figure 15:**
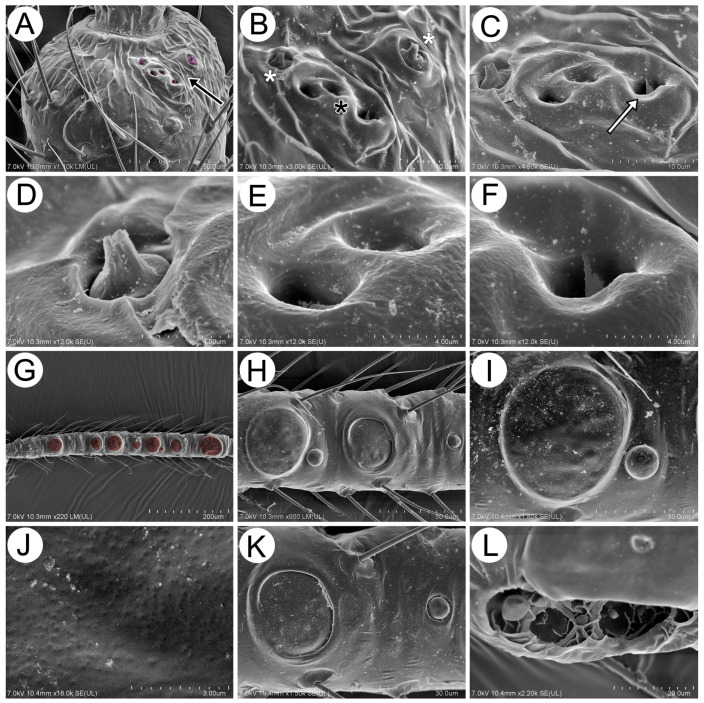
SEM of antennal sensilla of alate viviparous females of *M. sorini*: (**A**) ventral side of the pedicel with many rhinariola (arrow), (**B**,**C**) structure of two general kinds of rhinariola, exposed (white asterisks) and sunken (black asterisks), one with a visible projection (arrow), (**D**–**F**) fine structure of all kinds of exposed and sunken rhinariola, (**G**) ANT III with trichoid sensilla and small placoid sensilla, (**H**) different sized small placoid sensilla, (**I**–**K**) fine structure of different sized small placoid sensilla and porous surface, and (**L**) accessory rhinaria on ANT VI.

In the alate viviparous females, five rhinariola on the pedicel were visible (Figure 15A). There are two kinds of rhinariola; they are arranged in two groups and are similar to sunken coeloconic sensilla on the basal part of ANT VI. Two rhinariola of type A are exposed with visible projections, and three of type B are deeply located (Figure 15B,C). Rhinariola type A is characterized by a button-shaped base (3–4 μm in diameter) and a cone-shaped apex, formed from about 4–6 projections. This kind of rhinariolum is visibly separated from the cavity in which it is sited (Figure 15D). The remaining three rhinariola of type B are in the form of holes, 2–4 μm in diameter, and only in one of them do two finger-like projections extend over the hole (Figure 15E,F). The antennal segment III of alate viviparous females bears multiporous placoid sensilla, which are arranged in one regular row along the whole length of the segment and are of two sizes and differently nested (Figure 15G,H). Most of the placoid sensilla are big, almost as wide as the segment, 28–57 μm in diameter, while very few are small, 9–10 μm in diameter and 10–12 pores per μm^2^ (Figure 15J). All sensilla may be slightly protuberant or lie in very flat, narrow cavities with very smooth edges (Figure 15I,K). In alate viviparous females, the sensilla on the last antennal segment are the same as in apterous viviparous females (Figure 15L).

#### 3.2.4. Sensilla of the Legs and on the Wings

**Figure 16:**
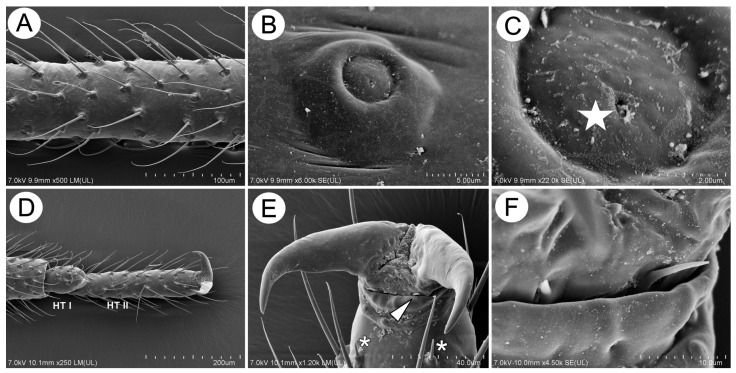
SEM of sensilla on the legs of *M. sorini*: (**A**) surface and trichoid sensilla on hind tibiae, (**B**,**C**) structure of campaniform sensilla on femora and HT II with small pore (star) near the edge, (**D**) structure and trichoid sensilla of the hind tarsus, (**E**) distal part of HT II with two very short trichoid sensilla on the ventral side (asterisks) and a very short parempodia (arrowhead), and (**F**) fine structure of the very short and pointed parempodia.

**Figure 17:**
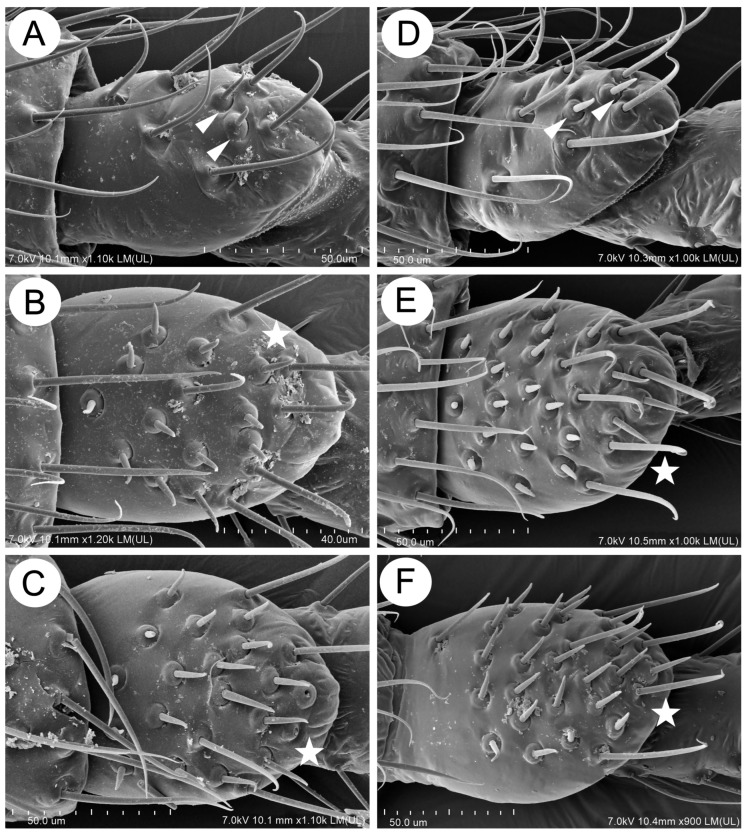
SEM of the first segments of the tarsi of *M. sorini*: (**A**) HT I of the aptera with two sense pegs (arrowheads), (**B**) MT I of the aptera with sense pegs on almost the whole surface of the ventral side and normal trichoid sensilla on the distal end (star), (**C**) FT I of the aptera with sense pegs on the whole surface of the ventral side and normal trichoid sensilla on the distal end (star), (**D**) HT I of alata with two sense pegs (arrowheads), (**E**) MT I of alata with sense pegs on almost the whole surface of the ventral side and normal trichoid sensilla on the distal end (star), and (**F**) HT I of alata with sense pegs on the whole surface of the ventral side and normal trichoid sensilla on the distal end (star).

**Figure 18:**
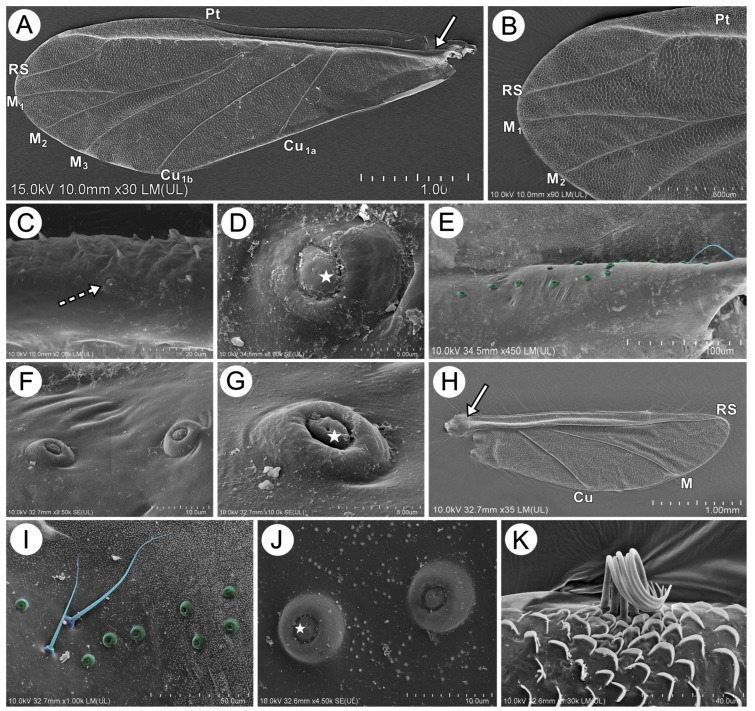
SEM of the wing structure and sensilla of alate viviparous females of *M. sorini*: (**A**) fore wing, (**B**) structure of the distal part of fore wing, (**C**,**D**) campaniform sensilla on the Sc + R+M + Cu (dotted arrow) with a pore in the central point (star), (**E**) numerous campaniform sensilla on the proximal part of fore wing near the wing articulation, (**F**,**G**) structure of campaniform sensilla from the wing articulation area with a pore in central point (star), (**H**) hind wing with a group of sensilla on the proximal part (arrow), (**I**) campaniform and trichoid sensilla in the wing articulation area, (**J**) fine structure of campaniform sensilla on hind wings with pores closer to the edge (star), and (**K**) connecting area on hind wing strengthened with numerous lamina and six hooks: Pt—pterostigma, Rs—radial sector, M_1_, M_2_, M_3_—media, and Cu1a and Cu1b—cubitus.

The legs, like the rest of the body and antennae, are covered with numerous long, fine, pointed trichoid sensilla with rounded, slightly protuberant sockets (Figure 16A). The tarsi are two-segmented and covered with many trichoid sensilla (Figure 16D). Additionally, on the dorsal side of HT II, one campaniform sensillum is visible. The sensillum is rounded, slightly protuberant, and formed by two subunits: the bigger collar is 10–13 μm in diameter, and the inner oval cap is about 5 μm long and about 4 μm, with a small, narrow oval pore near the edge, about 0.32 μm (Figure 16B,C). The pretarsus is normal, with two parempodia (empodial setae) and two claws with single pointed, curved apices (Figure 16E). The parempodia are poorly developed, about 6–7 μm long, and pointed (Figure 16F). The first segment of the tarsi is characterized by numerous short, rigid trichoid sensilla with more protuberant and more circular sockets than the very long, hair-like trichoid sensilla—sense pegs. The first segment of the hind tarsi of apterous and alate viviparous females carry two sense pegs (Figure 17A,D). The first segments of the middle tarsi have about 12 sense pegs in apterous and 19 in alate viviparous females (Figure 17B,E). The first segments of the fore tarsi bear about 17 sense pegs in apterous and 21 in alate viviparous females (Figure 17C,F). The wings are normal-shaped, with slightly curved Cu_1b_, twice-branched media radial sector running down (Figure 18A,B). The inner side of the Sc + R + M + Cu vein bears many campaniform sensilla, which are very similar to those on HT II, about 11–12 μm in diameter of the collar, but the pore is located in the central part of the cap, not near the edge (Figure 18C,D). On the basal part of the wings (near the wing articulation), campaniform sensilla are more numerous, about 15–17 (Figure 18E), and slightly smaller (8–9 μm in diameter of the collar) than those on the vein (Figure 18F,G). The hind wings (Figure 18H) have campaniform sensilla mostly on the basal part, about 8–10 in number, with two additional long, fine, pointed trichoid sensilla (Figure 18I). Campaniform sensilla on the hind wing are more rounded and more protuberant than those on the fore wings. Their collar is about 7 μm in diameter, and the circular cap is about 2.70–2.80 μm in diameter. Their pores are not so central as in campaniform sensilla on the fore wings (Figure 18J). The claval area is characterized by many densely lying scale-like elements and about 6–7 hamuli (Figure 18K).

#### 3.2.5. Morphology of Sexual Morphs and Eggs

**Figure 19:**
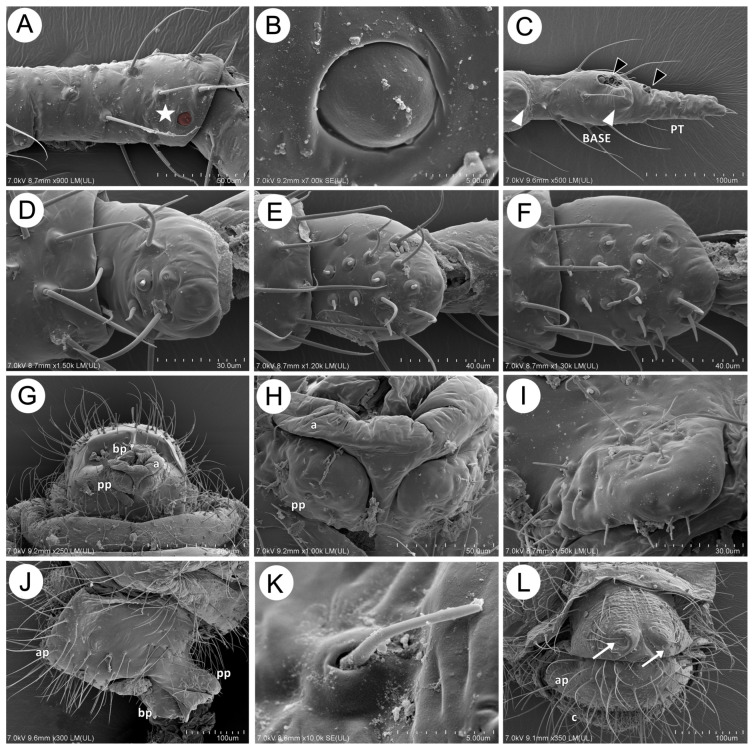
SEM of the morphological characters of the apterous male of *M. sorini*: (**A**) trichoid and small multiporous placoid sensilla (star) on ANT IV, (**B**) fine structure of the multiporous placoid sensillum, (**C**) ANT VI rhinaria with accessory rhinaria (black arrowhead) separated into two groups, on the PT and near the major rhinarium (white arrowhead), (**D**) HT I sense pegs, (**E**) MT I sense pegs, (**F**) FT I sense pegs, (**G**) ventral side of genitalia, (**H**,**I**) structure and sensilla of parameres, (**J**) lateral side of genitalia, (**K**) fine structure of trichoid sensillum on parameres, and (**L**) genitalia buds (arrows) in the last instar larvae, a–aedeagus, ap–anal plate, bp–basal part of phallus, pp–parameres, c–cauda.

The apterous male and oviparous females manifest most of the described characters and sensilla as in the parthenogenetic generation. All antennal sensilla are very similar to those in apterous or alate viviparous females, except that the placoid sensilla on the antennal flagellum (secondary rhinaria) are smaller (8–9 μm in diameter) and slightly embedded, with clearly visible pores, 15–20 per μm^2^ (Figure 19A,B). The sensilla on the last antennal segment are similarly located as in parthenogenetic females, with one separate small placoid sensillum (one of the accessory rhinaria) on the basal part of the terminal process (Figure 19C). The first segments of the hind, middle, and fore tarsi also are characterized by numerous “sense pegs”, FT I with 15, MT I with 10, and HT I with 2 (Figure 19D–F). The male genitalia are rather similar to others known in the Lachninae. The parameres are clearly visible and separate, located directly under the basal part of the phallus (Figure 19G,H). They are characterized by semi-circular apices and covered with many short, rigid, slightly pointed trichoid sensilla, about 5–12 μm long, in well-developed, circular sockets. The basal part of the phallus is characterized by rather wide, non-elongated apices (Figure 19I–K). Genital buds are clearly visible in male larvae as two protuberant papilla-like structures, covered with short, rigid trichoid sensilla (Figure 19L).

**Figure 20:**
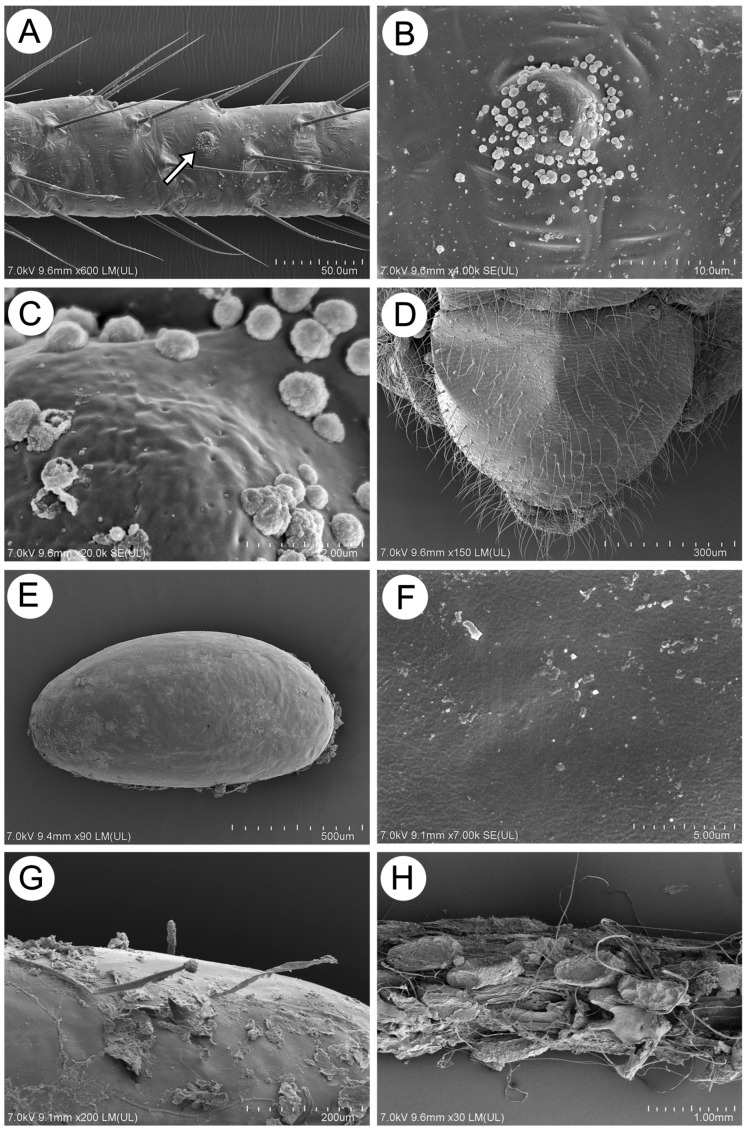
SEM of the morphological characters of oviparous females and eggs of *M. sorini*: (**A**) part of the hind tibia with many setae and visible pseudosensorium (arrow), (**B**) structure of the pseudosensorium, (**C**) fine structure of the pseudosensorium and secretions, (**D**) genital plate, (**E**) egg general view, (**F**) fine structure of the egg surface, (**G**) egg surface with fiber, and (**H**) eggs on a shoot with many secretions.

Oviparous females are especially characterized by scant, poorly visible scent plaques (Figure 20A). A single scent plaque is a protuberant and porous area of ill-defined borders, about 9–10 μm in diameter, with numerous very small, granulose secretions (Figure 20B,C). The subgenital plate is large, wider in the lateral than in the distal part, and covered with numerous long, very fine, pointed setae and minute spinules (Figure 20D). Eggs are about 1.10–1.13 mm long and 0.60–0.65 mm wide, smooth, and covered with thin fibers produced by the oviparous females (Figure 20E–H).

### 3.3. Biology of Miyalachnus

**Figure 21:**
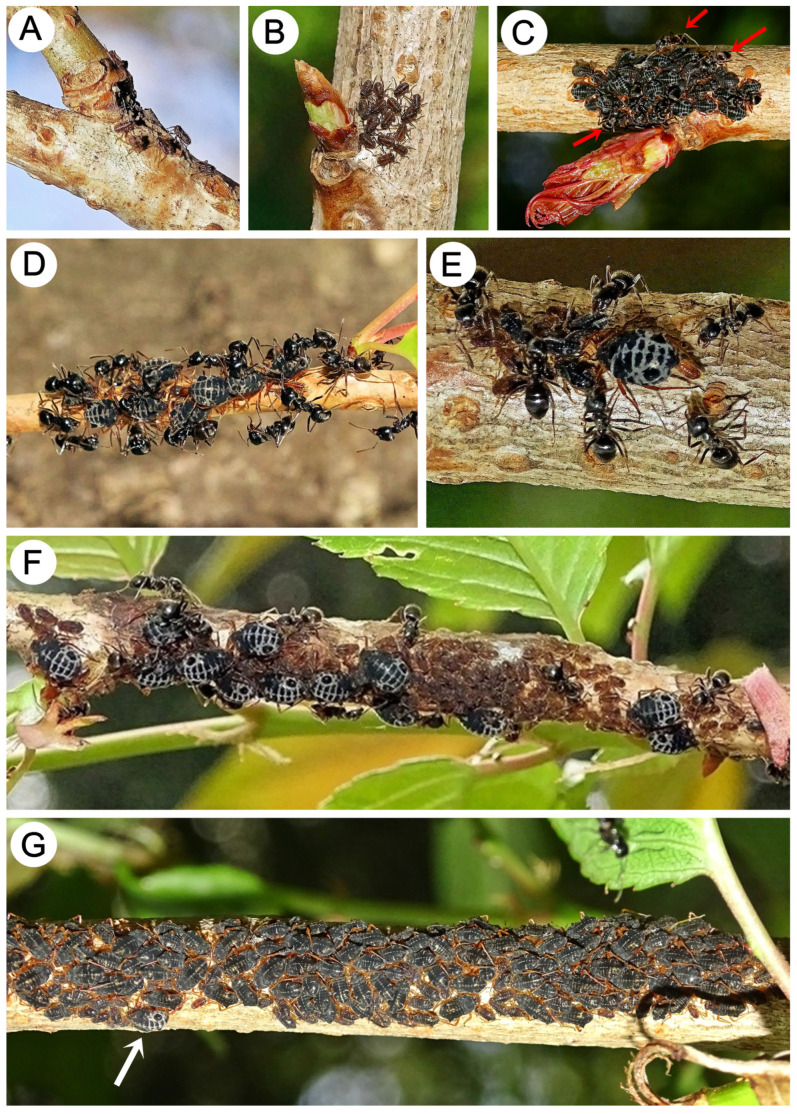
Biology of *M. sorini* (fundatrices and nymphs): (**A**) freshly hatched fundatrices’ larvae, (**B**) fundatrices’ larvae in a small colony near the leaf bud, (**C**) last instar larvae and adult fundatrices in a small colony near the leaf bud with ants (red arrows), (**D**) adult fundatrices under the protection of *L. japonicus* start to give birth to fundatrigeniae, (**E**) adult fundatrix giving birth to fundatrigenia larvae, (**F**) colony of adult fundatrices and first instar nymphs visited by *L. japonicus*, and (**G**) large colony of nymphs of alate viviparous females (fundatrigeniae) and one of the last fundatrices (arrow).

In Rokuto (Ibaraki Prefecture), where the main observations were made, the fundatrices’ larvae were observed on 16 February for the first time, and they had already hatched. The first instars were observed near the egg capsules, and some of them were moving slowly (Figure 21A). At the same time, a small colony of 15 larvae was observed. These were distinctly larger than the few mentioned in the egg area, probably second instar (Figure 21B). The fundatrices become adults probably after a long time, as at the end of March (25.03), the last instars and colonies of adult stem mothers were observed. When they became adults, they began to be attended by ants (Figure 21C). At the beginning of April, adult fundatrices in small colonies of about 7–12 individuals began to give birth to the first instars of viviparous females (fundatrigeniae). At the same time, ants that were visiting the colonies started to be more numerous (Figure 21D,E). On 18 April, in the colonies, adult fundatrices and numerous first instars of larvae were observed (Figure 21F). The larvae that were born by the stem mothers were all larvae of alate viviparous females, and on 25 and 28 April, large colonies of alate viviparous female nymphs and very few fundatrices were found (Figure 21G).

**Figure 22:**
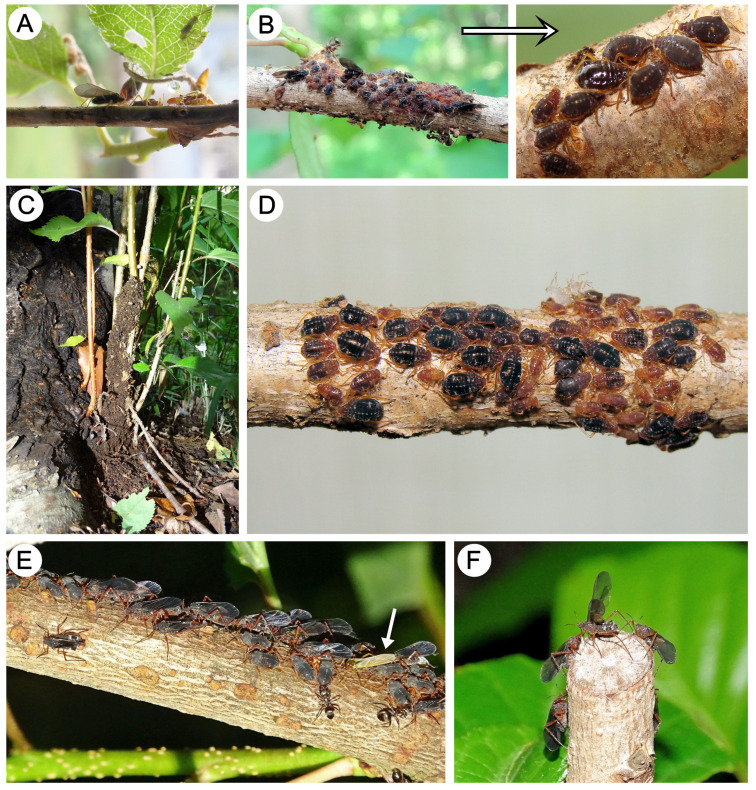
Biology of *M. sorini* (apterous and alate viviparous females): (**A**) an alate viviparous female with larvae, (**B**) colony of apterous and alate viviparous females and recently molted apterous viviparous females with shiny and without greyish markings, (**C**) colony of young shoots near the ground, covered by soil shelters, (**D**) colony of summer apterous viviparous females after removing the soil, (**E**) colony of last instar nymphs and alate viviparous females with freshly molted alata (arrow), and (**F**) alate viviparous females during preparation for migration.

The alate viviparous females become adults probably at the beginning of May; on 7 May, small colonies of alate viviparous females with larvae of apterous viviparous females were observed (the apterous viviparous females were not offspring of the fundatrices) (Figure 22A). On 19 May, colonies of alate viviparous females, adult apterous viviparous females, and their larvae or only apterous females were observed (Figure 22B). All generations fed on branches of *Prunus* trees. At the end of May, in most colonies, the apterous viviparous females predominated, and if they fed on young shoots near the ground, they were hidden and protected by ants in the form of soil shelters (Figure 22C,D). Also, at the same time (mid-May), the last colonies of alate viviparous females flew to colonize other trees (Figure 22E,F).

**Figure 23:**
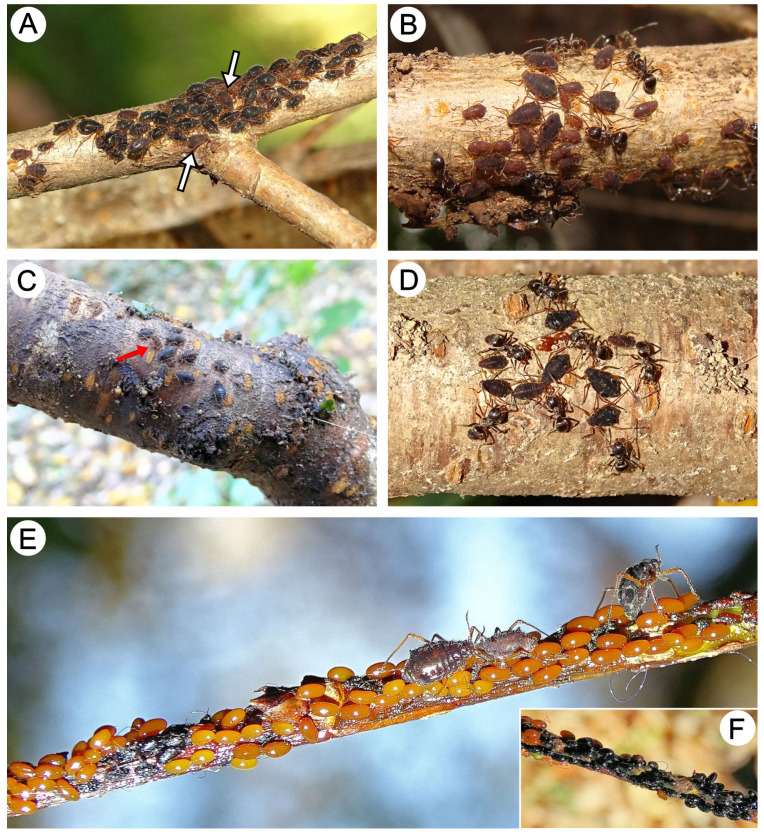
Biology of *M. sorini* (sexuparae and sexuales): (**A**) colony of apterous viviparous females with appearing sexuparae (white arrows), (**B**) autumnal colony of sexuparae and sexuales larvae, (**C**) oviparous females and male (red arrow), (**D**) oviparous females start laying eggs, (**E**) freshly laid eggs, and (**F**) several day-old eggs.

At the end of summer and the beginning of autumn (late September), many morphs in the colonies began to change in sexuparae, which differ from apterous viviparous females in pigmentation (they lack the white wax markings) (Figure 23A). From 13 October to the end of the month, only colonies of these morphs were observed (Figure 23B). Sexuparae and the last apterous viviparous females were observed in early November (7.11), and on 22 November, adult sexual morphs (oviparous females and apterous males) were observed (Figure 23C). At the same time, sexuales began to copulate, and the first oviparous females started laying eggs (Figure 23D). Egg laying lasted until mid-December (Figure 23E) when only black winter eggs were observed on the branches (Figure 23F). *Miyalachnus sorini* representatives were visited by *Lasius japonicus*.

## 4. Discussion

Based on Sorin’s [26] description of *P*. *imbricatus nipponicus* it is evident that the Japanese subspecies differs significantly from the nominate species across several traits, leading Blackman and Eastop [27] to confer full species status. Blackman and Eastop [27] likely considered the notably smaller size of the Japanese taxon, along with the positional shift of one accessory rhinarium to the PT, as the main diagnostic features highlighted by Sorin [26]. Upon examining type specimens of both *P*. *imbricatus* subspecies, we observed that the Indian *P*. *imbricatus imbricatus* more closely resembles other *Pyrolachnus* species in body size and general morphology. However, the extensively imbricated dorsal surface of the thorax and abdomen, along with thick setae, makes this species remarkably distinct from its *Pyrolachnus* congeners.

*Pyrolachnus imbricatus nipponicus* not only differed in the previously described traits (body size and accessory rhinaria arrangement) but also exhibited a dorsal sculpture with abundant, well-developed denticles lacking in *P*. *imbricatus imbricatus* (and other *Pyrolachnus* species). Additionally, apterous and alate viviparous females possessed significantly more sense pegs on the fore and middle tarsi (even exceeding *Sinolachnus*) than any other Lachninae members. The discovery of a second species, which differs from *P*. *imbricatus nipponicus* but shares some of the same characteristics, confirmed the suspicion that they are members of a new, previously undescribed genus. Considering the shared characters of generic features, *Miyalachnus* species differ from other Tuberolachnini and Lachninae genera, primarily by the uniformly brown wings of alate viviparous females, which are uncommon within the entire Lachninae, with some exceptions in *Sinolachnus*. The rest of the lachnids are characterized by hyaline wings (Eulachnini, *Indolachnus*, *Nippolachnus*, *Tuberolachnus salignus*, other species feeding on *Salix*, root-feeding Tramini, and some *Sinolachnus* and Stomaphidini species), maculated wings (Lachnini like *Lachnus* Burmeister, 1835, *Pterochloroides* Mordvilko, 1914, and *Maculolachnus* Gaumont, 1920, with only one dark spot on the membrane), or wings with only the basal part being brown (*Pyrolachnus* in Tuberolachnini and *Longistigma* Wilson, 1909 in Lachnini).

*Miyalachnus* members, along with *Sinolachnus* species, share the arrangement of accessory rhinaria on the last antennal segment (with one moved to the PT), distinguishing both genera easily from the rest of the Lachninae. Additionally, *Miyalachnus* alate viviparous females bear large, flat secondary rhinaria, which completely distinguish them from *Sinolachnus*, other Tramini, *Pyrolachnus*, *Tuberolachnus* Mordvilko, 1909, and *Stomaphis* Walker, 1870, where secondary rhinaria are much more numerous and extremely small, oval, or irregular in shape and often protuberant. Among *Nippolachnus* species, only the secondary rhinaria may appear somewhat similar, but in *Miyalachnus*, they are noticeably smaller, rounder, and flatter compared to those in *Nippolachnus*.

On the other hand, numerous morphological and biological similarities can be noted between *Miyalachnus* and *Sinolachnus*, and the new genus undoubtedly has some close relationships with *Sinolachnus* and maybe Tramini, with some evident exceptions, of course. Most distinctly, *Miyalachnus* is set apart not only from *Sinolachnus* but from the entire Lachninae subfamily by having the highest number of sense pegs on the first tarsal segments of the fore and middle legs. *Sinolachnus* has the second-highest count for this trait, and Tramini representatives also possess many sense pegs (4–6 on the fore tarsi). The arrangement of accessory rhinaria in *Miyalachnus* closely resembles that in *Sinolachnus*—one of the two small multiporous placoid sensilla is separated from the other accessory rhinaria (the second placoid sensillum and four sunken coeloconic sensilla) and lies on the terminal process. Moreover, apart from *Miyalachnus* and *Sinolachnus*, such partial shifting of accessory rhinaria is known only in certain Tramini (*Trama* von Heyden, 1837 and *Protrama* (Baker, 1920)) and *Nippolachnus* (Tuberolachnini) across all Lachninae and most Aphidinae.

One distinctive and previously neglected feature in Lachninae, the number and length of the “sense pegs” (or peg-like setae) on the ventral side of the first segments of the fore, middle, and hind tarsi of the legs, has been shown to be important in different areas of research, both taxonomical and phylogenetic. As a result of detailed analyses of this feature in Tuberolachnini and other members of the Lachninae, the number range and the length of sense pegs are constant between genera and tribes. In the case of Tuberolachnini, we can observe two separate groups. *Nippolachnus* is the most distinctive, with one sense peg [1-1-1] on the first segments of the fore, middle, and hind tarsi, whereas *Tuberolachnus* is characterized by [(2-3)-(2-3)-(2)] sense pegs, and *Pyrolachnus* has [(4-6)-(4-6)-(3-4)] of these sensilla. *Sinolachnus* differs from other Tuberolachnini members in many morphological characteristics. The first one is the secondary rhinaria of alate viviparous females, which are extraordinary within the Aphididae due to their great number (from several dozen to greatly exceeding a hundred on one segment); they are also very small and protuberant (also quite an uncommon feature in aphids), and regarding this characteristic, they are more similar to those in *Eotrama* (Hille Ris Lambers, 1969) and *Protrama*. Also, the presence of secondary rhinaria on the antennae (often on the last segment) of the apterous morphs makes *Sinolachnus* more closely related to Tramini than Tuberolachnini. Finally, *Sinolachnus* differs from the rest of the genera by having many more numerous sense pegs on the first segments of the tarsi (7–9 on FT I and 5–7 on MT I), making it more similar to Tramini than other Tuberolachnini. In general, *Sinolachnus* is clearly one of the most challenging Lachninae genera, and perhaps, this was the reason that in some cases, species from this genus have been described in other genera, such as *Maculolachnus* or *Cinara* Curtis, 1835. Kanturski et al. [23], during the revision of the genus, pointed out several differences between *Sinolachnus* from Tuberolachnini and similarities with Tramini, transferring it to the latter tribe.

Sorin [26] probably did not have the chance to examine the type material of *P*. *imbricatus imbricatus*, and the superficial similarity (from the descriptions) and host plant of both *Miyalachnus* and *P*. *imbricatus* (*Prunus*) led him to place this species within *Pyrolachnus*. As we have demonstrated above, *Miyalachnus nipponicus* and *M*. *sorini* are not conspecific with *P*. *imbricatus imbricatus*, nor are they congeneric with *Pyrolachnus*, and their characteristics, in contrast to all previously known Lachninae genera, justifying the establishment of a separate genus. In the future, the collection of the unusually difficult-to-obtain *Eotrama* will definitely and finally solve the questions about the relationships between *Miyalachnus*, *Sinolachnus*, and the remaining Tramini genera.

## 5. Conclusions

The detailed and comparative morphological evidence suggests that a taxonomic revision is warranted for numerous Lachninae taxa, particularly in Tuberolachnini, and this should include *Eotrama* representatives in forthcoming molecular analyses. This endeavor will undoubtedly shed light on the relationships between Tuberolachnini, *Miyalachnus*, *Sinolachnus*, and Tramini, which are the first author’s ongoing work.

## Figures and Tables

**Figure 8:**
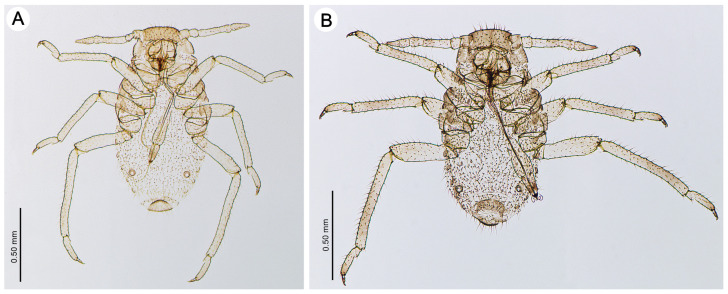
First instar larvae of the genus *Miyalachnus*: (**A**) *M. nipponicus* and (**B**) *M. sorini*.

**Table 1:** Morphological differences between apterous and alate viviparous females of *Miyalachnus* gen. nov., *Pyrolachnus*, and *Sinolachnus*.

Character	*Miyalachnus* gen. nov.	*Pyrolachnus*	*Sinolachnus*
	Apterous Viviparous Females		
BL	2.85–3.30	4.25–5.20	2.50–3.50
HW/ANT	0.53–0.60	0.41–0.53	-
ANT IV	0.12–0.17	0.23–0.34	0.20–0.24
ANT V	0.15–0.17	0.20–0.30	0.21–0.24
PT/BASE	0.73–0.96	0.48–0.66	0.50–0.60
URS/ANT III	0.35–0.43	0.20–0.28	0.31–0.40
URS/HT II	0.58–0.68	0.43–0.51	about 0.85
FT I pegs	13–21	4–6	6–12
MT I pegs	8–14	3–5	2–5
ANT VI	Without secondary rhinaria. Accessory rhinaria are divided into two groups. One single, situated separately over the major rhinarium on the PT. The remaining accessory rhinaria tightly adhere to each other and lie next to the major rhinarium. PT tapering from the basal part.	Without secondary rhinaria. Accessory rhinaria in one group, tightly adhering to each other under or next to the major rhinarium. PT tapering progressively towards the apex.	With secondary rhinaria. Accessory rhinaria in one group tightly adhere to each other next to the major rhinarium. PT tapering from the basal part.
	Alate viviparous females		
Antennae	With typical secondary rhinaria in the form of big, round plates, mostly in one row on ANT III and IV. The accessory rhinaria scheme on ANT VI is like in apterae.	With typical secondary rhinaria in the form of big, round plates, mostly in one row on ANT III, IV, and V. The accessory rhinaria scheme on ANT VI is like in apterae.	Secondary rhinaria in the form of numerous (up to 400) small protuberant sensilla on ANT III–VI. The accessory rhinaria scheme on ANT VI is like in apterae.
Wings membrane	Wings uniformly light brown to brown. Uniformly covered by membrane lamina.	Wings brownish only at basal part (not exceeding Cu_1a_). The rest of the wing is hyaline. Membrane lamina from the mid to the apical part.	Wings uniformly hyaline. Membrane lamina from the mid to the apical part.
Pterostigma	Medium in length. Not longer than 6.70 times the width.	Long and narrow, up to 10.90 times longer than the width.	Short. Not longer than 5.00 times the width.
Media	Twice branched. M_1_ and M_2_ as long as or shorter than Rs.	Twice branched. M_1_ and M_2_ longer than Rs.	Once or twice branched. When once branched, M_1_ and M_2_ are longer than Rs. When twice branched, M_1_ and M_2_ are shorter than Rs.
FT I pegs	10–20	4–6	2–9
MT I pegs	11–15	4–5	2–6

**Table 2:** Measurements (in mm) of known morphs and first instar larva of *Miyalachnus nipponicus*. * in the first instar larva ANT 5-segmented.

Character	*Miyalachnus nipponicus*			
	Fundatrix	Apterous viv. fem.	Alate viv. fem.	First Instar Larva
BL	3.55–3.75	2.85–3.30	3.78–3.80	1.25–1.55
Max W	2.37–2.75	1.70–2.10	1.80	-
HW	0.71–0.76	0.66–0.74	0.72–0.78	0.50–0.54
ANT	1.22–1.30	1.18–1.33	1.44–1.62	0.58–0.60
ANT III	0.49–0.50	0.43–0.51	0.58–0.65	0.17
ANT IV	0.13–0.14	0.12–0.14	0.18–0.21	0.10
ANT V	0.14–0.16	0.15–0.17	0.19–0.22	-
ANT VI (V *)	0.20–0.23	0.21–0.25	0.25–026	0.15
BASE	0.13–0.15	0.11–0.13	0.15–0.18	0.06
PT	0.07–0.08	010–0.12	0.07–0.10	0.09
III FEMORA	1.25–1.32	1.04–1.22	1.60–1.62	0.92–1.00
III TIBIAE	2.12–0.30	1.84–2.20	2.85–2.92	1.67–1.82
HT I basal L	0.05	0.04–0.05	0.05	-
HT I dorsal L	0.02–0.03	0.02–0.03	0.02–0.03	-
HT I ventral L	0.11–0.12	0.10–0.11	0.12–0.13	0.07
HIT I intersegmental L	0.08–0.09	0.08	0.09	-
HT II	0.30–0.32	0.27–0.31	0.36–0.37	0.20–0.21
URS	0.16–0.17	0.18–0.19	0.20–0.21	0.16
SIPH sclerite	0.63–0.69	0.52–0.63	0.45–0.47	0.13–0.15
Genital plate length	0.26–0.28	0.30–0.40	0.33–0.35	-
Genital plate width	0.61–0.62	0.55–0.60	0.54–0.56	-
ANT III rhinaria	1	0–1	8–10	-
ANT IV rhinaria	1	0–2	1–2	-
ANT V rhinaria	-	1	1–3	-
Wing length	-	-	4.80	-

**Table 3:** Morphometric and morphological differences between apterous and alate viviparous females of *Miyalachnus nipponicus* and *M. sorini* sp. nov.

*Miyalachnus nipponicus*	*Miyalachnus sorini*
Fundatrices	
BL 3.55–3.77 URS with 15 accessory setae	BL3.80–4.22 URS with 9–10 accessory setae
LS/BD III 2.28–2.83	LS/BD III 1.62–2.00
HT Ib/HT Id 1.66–1.85	HT Ib/HT Id 2.20–2.75
HT Ib/HT Iv 0.40–0.43	HT Ib/HT Iv 0.45–0.50
HT Id/HT Ii 0.27–0.31	HT Id/HT Ii 0.22–0.25
Appendages uniformly yellow to brown	Appendages with darker distal parts
ABD II–IV without sclerites or scleroites	ABD II–IV with sclerites and scleroites
Apterous viviparous females	
URS with 15–18 accessory setae PT/BASE 0.90–0.96	URS with 9–10 accessory setae PT/BASE 0.73–0.84
Hind leg/BL 1.05–1.17	Hind leg/BL 1.23–1.34
URS/HT II 0.62–0.68	URS/HT II 0.58–0.60
Appendages uniformly yellow to light brown	Appendages with darker distal parts
Abdomen membranous with poorly developed sclerotization	Abdomen strongly sclerotized in the form of sclerites, cross-bands, or solid shields.
URS straight	URS triangular
Genital plate in the form of one solid sclerite	Genital plate in the form of two separate sclerites
Alate viviparous females	
URS with 15–18 accessory setae PT/BASE 0.40–0.66	URS with 10–11 accessory setae PT/BASE 0.70–0.71
URS/ANT VI 0.75–0.83	URS/ANT VI 0.64–0.71
HT II/ANT VI 1.39–1.46	HT II/ANT VI 1.21–1.32
ANT VI/ANT III 0.40–0.43	ANT VI/ANT III 0.45–0.48
FT I sense pegs 10–11	FT I sense pegs 18–20
Common node of M1 and M2 under the beginning of the radial sector	Common node of M1 and M2 under the tip of the pterostigma
First instar larvae	
HW/ANT 0.85–0.90	HW/ANT 0.79–0.80
PT/BASE 1.38–1.50	PT/BASE 1.21–1.26
LS/BD III 1.71–1.75	LS/BD III 1.10–1.37
III/BL 1.80–2.41	III/BL 0.81–0.92

**Table 4:** Measurements (in mm) of known morphs and first instar larva of *Miyalachnus sorini* sp. nov. * in first instar larva ANT 5-segmented.

Character	*Miyalachnus sorini*					
	Fundatrix	Apterous viv. fem.	Alate viv. fem.	Oviparous Female	Apterous Male	First Instar Larva
BL	3.80–4.22	3.00–3.25	3.75–4.02	2.75–3.40	2.00–2.05	1.37–1.42
Max W	2.85–2.87	1.75–2.05	1.87–2.00	1.65–1.90	1.00–1.02	-
HW	0.78–0.79	0.70–0.76	0.75–0.77	0.60–0.70	0.57–0.58	0.48–0.49
ANT	1.30–1.41	1.23–1.37	1.47–1.53	1.06–1.29	0.94–1.06	0.60–0.61
ANT III	0.51–0.58	0.44–0.49	0.56–0.60	0.38–0.52	0.33–0.38	0.17–0.18
ANT IV	0.13–0.15	0.14–0.17	0.19–0.20	0.11–0.15	0.10–0.12	0.10–0.11
ANT V	0.16–0.17	0.16–0.17	0.20–0.21	0.14–0.17	0.12–0.14	-
ANT VI (V *)	0.23–0.24	0.23–0.26	0.26–0.28	0.22–0.23	0.21–0.22	0.15–0.17
BASE	0.15–0.16	0.12–0.15	0.15–0.16	0.12–0.13	0.10–0.11	0.07
PT	0.08	0.10–0.11	0.11–0.12	0.09–0.10	0.10–0.12	0.08–0.09
III FEMORA	1.27–1.37	1.30–1.40	1.65–1.67	1.02–1.25	0.69–0.75	0.37–0.9
III TIBIAE	2.12–2.35	2.25–2.37	2.85–2.90	1.80–2.25	1.17–1.22	0.52–0.71
HT I basal L	0.05	0.04–0.05	0.04–0.05	0.04–0.05	0.04	-
HT I dorsal L	0.02	0.02–0.03	0.02–0.03	0.01–0.02	0.01–0.02	-
HT I ventral L	0.11–0.12	0.10–0.12	0.11–0.12	0.10–0.11	0.08–0.09	0.07–0.08
HIT I intersegmental L	0.09–0.10	0.08–0.09	0.08–0.09	0.07–0.08	0.06	-
HT II	0.31–0.34	0.30–0.31	0.33–0.35	0.25–0.28	0.24–0.25	0.21–0.22
URS	0.18–0.20	0.17–0.18	0.18–0.19	0.16–0.17	0.16–0.17	0.17
SIPH sclerite	0.70–0.80	0.65–0.70	0.50–0.57	0.60–0.90	0.14–0.20	0.06–0.07
Genital plate length	0.30–0.35	0.35–0.40	0.28–0.30	0.45–0.50	-	-
Genital plate width	0.66–0.70	0.68–0.72	0.56–0.60	0.60–0.70	-	-
Wing length	-	-	4.45–4.67	-	-	-

## Data Availability

All data generated or analyzed during this study are included in this published article.
